# From Proteomic Mapping to Invasion-Metastasis-Cascade Systemic Biomarkering and Targeted Drugging of Mutant BRAF-Dependent Human Cutaneous Melanomagenesis

**DOI:** 10.3390/cancers13092024

**Published:** 2021-04-22

**Authors:** Aikaterini F. Giannopoulou, Athanassios D. Velentzas, Athanasios K. Anagnostopoulos, Adamantia Agalou, Nikos C. Papandreou, Stamatia A. Katarachia, Dimitra G. Koumoundourou, Eumorphia G. Konstantakou, Vasiliki I. Pantazopoulou, Anastasios Delis, Maria T. Michailidi, Dimitrios Valakos, Dimitris Chatzopoulos, Popi Syntichaki, Vassiliki A. Iconomidou, Ourania E. Tsitsilonis, Issidora S. Papassideri, Gerassimos E. Voutsinas, Polydefkis Hatzopoulos, Dimitris Thanos, Dimitris Beis, Ema Anastasiadou, George Th. Tsangaris, Dimitrios J. Stravopodis

**Affiliations:** 1 Section of Cell Biology and Biophysics, Department of Biology, School of Science, National and Kapodistrian University of Athens (NKUA), 15701 Athens, Greece; aigiann@biol.uoa.gr (A.F.G.); tveletz@biol.uoa.gr (A.D.V.); npapand@biol.uoa.gr (N.C.P.); skatarachia@biol.uoa.gr (S.A.K.); dim.koum99@gmail.com (D.G.K.); marymichailidi@gmail.com (M.T.M.); veconom@biol.uoa.gr (V.A.I.); ipapasid@biol.uoa.gr (I.S.P.); 2 Systems Biology Center, Biomedical Research Foundation of the Academy of Athens (BRFAA), 11527 Athens, Greece; atanagnost@bioacademy.gr (A.K.A.); gthtsangaris@bioacademy.gr (G.T.T.); 3 Center for Clinical, Experimental Surgery and Translational Research, Biomedical Research Foundation of the Academy of Athens (BRFAA), 11527 Athens, Greece; agalou@bioacademy.gr (A.A.); dbeis@bioacademy.gr (D.B.); 4 Massachusetts General Hospital Cancer Center (MGHCC), Harvard Medical School, Charlestown, Boston, MA 02114, USA; ekonstantakou@mgh.harvard.edu; 5 Center of Basic Research, Biomedical Research Foundation of the Academy of Athens (BRFAA), 11527 Athens, Greece; vaspantazo@bioacademy.gr (V.I.P.); tdelis@bioacademy.gr (A.D.); dvalakos@bioacademy.gr (D.V.); dchatzop@bioacademy.gr (D.C.); synticha@bioacademy.gr (P.S.); thanos@bioacademy.gr (D.T.); anastasiadou@bioacademy.gr (E.A.); 6 Section of Animal and Human Physiology, Department of Biology, School of Science, National and Kapodistrian University of Athens (NKUA), 15701 Athens, Greece; rtsitsil@biol.uoa.gr; 7 Laboratory of Molecular Carcinogenesis and Rare Disease Genetics, Institute of Biosciences and Applications (IBA), National Center for Scientific Research (NCSR) “Demokritos”, 15310 Athens, Greece; mvoutsin@bio.demokritos.gr; 8 Department of Biotechnology, Agricultural University of Athens (AUA), 11855 Athens, Greece; phat@aua.gr

**Keywords:** biomarker, BRAF, cancer, IMC, LC-MS/MS, melanoma, metastasis, proteomics, WM115, WM266-4

## Abstract

**Simple Summary:**

Despite the recent advances in human malignancy therapy, metastasis and chemoresistance remain the principal causes of cancer-derived deaths. Given the fatal forms of cutaneous metastatic melanoma, we herein employed primary (WM115) and metastatic (WM266-4) melanoma cells, both obtained from the same patient, to identify novel biomarkers and therapeutic agents. Through state-of-the-art technologies including deep proteome landscaping, immunofluorescence phenotyping, and drug toxicity screening, we were able to describe new molecular programs, oncogenic drivers, and drug regimens, controlling the invasion-metastasis cascade during BRAF^V600D^-dependent melanomagenesis. It proved that proteomic navigation could foster the development of systemic biomarkering and targeted drugging for successful treatment of advanced disease.

**Abstract:**

Melanoma is classified among the most notoriously aggressive human cancers. Despite the recent progress, due to its propensity for metastasis and resistance to therapy, novel biomarkers and oncogenic molecular drivers need to be promptly identified for metastatic melanoma. Hence, by employing nano liquid chromatography-tandem mass spectrometry deep proteomics technology, advanced bioinformatics algorithms, immunofluorescence, western blotting, wound healing protocols, molecular modeling programs, and MTT assays, we comparatively examined the respective proteomic contents of WM115 primary (*n* = 3955 proteins) and WM266-4 metastatic (*n* = 6681 proteins) melanoma cells. It proved that WM115 and WM266-4 cells have engaged hybrid epithelial-to-mesenchymal transition/mesenchymal-to-epithelial transition states, with TGF-β controlling their motility in vitro. They are characterized by different signatures of SOX-dependent neural crest-like stemness and distinct architectures of the cytoskeleton network. Multiple signaling pathways have already been activated from the primary melanoma stage, whereas HIF1α, the major hypoxia-inducible factor, can be exclusively observed in metastatic melanoma cells. Invasion-metastasis cascade-specific sub-routines of activated Caspase-3-triggered apoptosis and LC3B-II-dependent constitutive autophagy were also unveiled. Importantly, WM115 and WM266-4 cells exhibited diverse drug response profiles, with epirubicin holding considerable promise as a beneficial drug for metastatic melanoma clinical management. It is the proteome navigation that enables systemic biomarkering and targeted drugging to open new therapeutic windows for advanced disease.

## 1. Introduction

Melanoma represents the deadliest form of skin cancer, with ~100,000 new incidents and ~7000 deaths estimated to have occurred for 2020 in the United States [1,2,3]. Its financial burden for clinical treatment remains cumbersome, since the approximate annual cost per patient ranges from $2000–$15,000 for stage I and $35,000–$155,000 for stage IV of the disease [2]. Among its strongest risk factors are family history, fair skin, multiple moles, immunosuppression, and ultraviolet radiation exposure [1,4]. For cutaneous thin melanoma, at the radial growth phase, surgical removal can afford a cure, whereas patients carrying tumors of intermediate thickness (Breslow depth; Clark level) (2–4 mm; from the upper epidermis layer to the innermost invasion depth) may eventually succumb to recurrence at regional or distant tissues [5,6]. Tumor thickness of 4 mm presents a high risk of metastasis and a median survival of 6–9 months [5]. Cells at the vertical growth phase feature growth-factor and anchorage independence, and presage distal metastasis [6]. Thickness-dependent metastasis is associated with genetic heterogeneity and therapy resistance of melanoma cell (sub-)populations [5,7].

Mutations in the *BRAF* gene comprise the most popular genetic aberrations in cutaneous melanoma, with an incidence range value of ~40–60% [2,4,5,6,7,8,9]. The glutamic acid for valine substitution at protein position 600 (V600E) represents ~80% of gene alterations and leads to ~500× upregulation of BRAF kinase activity that induces constitutive ERK-driven signaling in tumor cells [2,5,10,11]. Transition to invasive melanoma “inherits” driver mutations from the primary, early, cutaneous lesion(s) (e.g., *BRAF* and *TERT*) and “acquires” additional, critical, alterations, detrimentally deregulating cell-cycle control (e.g., *CDKN2A*; encodes p16^INK4A^ and p14^ARF^), cell identity (e.g., *ARID1A* or/and *ARID2*), cell growth (e.g., *PTEN*), and apoptosis (e.g., *TP53*) programs [7,12,13,14]. *PTEN* or *TP53* disabling mutations result in thicker invasive melanoma and advanced progression of the disease [7]. BRAF^V600E^ can cooperate with PTEN loss to generate metastatic melanoma, while lack of p16^INK4A^ may synergize with mutant, oncogenic, BRAF to induce metastasis [7,14,15]. Likewise, mutant p53 is able to accelerate BRAF^V600E^-orchestrated melanomagenesis, mechanistically evidencing the ultraviolet radiation-induced genotoxicity in human melanoma [16]. It is this mutational load and genomic heterogeneity that can fuel metastatic tumor cells with the advantage of resistance to therapy.

Treatment options for metastatic melanoma have advanced dramatically in the last ten years, with BRAF inhibitors (e.g., Vemurafenib and Dabrafenib), in mono-therapy or combination-therapy schemes, ameliorating patient survival and improving progression-free disease [17,18,19,20,21]. However, despite the initial clinical benefit, resistance against applied regimens will eventually develop [8,17,21,22,23]. Hitherto, described resistance mechanisms mainly include: (a) increased PDGFRβ (or IGF1R) expression [8,23,24,25], (b) NRAS (or MEKs) mutational activation [8,22,23,24], (c) dimerization of aberrantly spliced BRAF^V600E^ [8,23,26], (d) stroma cell-derived HGF secretion [23,27], (e) EGFR upregulation [25], (f) COT (kinase) amplification/activation [8,28], (g) MITF amplification/upregulation [23,29], and (h) loss of PTEN [8,30]. It may be an intratumor heterogeneity of resistance mechanisms that is linked to a mutational heterogeneity (multiple cell-specific molecular signatures) presumably residing in metastatic melanoma cells.

Metastasis represents the end product of a multistep cellular process termed the invasion-metastasis cascade (IMC) [31]. IMC is defined by the dissemination of “skillful” cancer cells from a primary tumor and their subsequent colonization in distant tissues [31,32,33]. This sequence of events involves cancer cell intravasation into the circulatory system, survival during hematogenous transit, arrest, extravasation through vascular wall into distant tissue parenchyma, micro-metastatic colony formation, and clinically (macroscopically) detectable, metastatic lesion growth (colonization) [31,33]. Hitherto, no gene mutation has proven to be characteristically associated with progression to metastasis. This indicates the need for prompt development of advanced systemic biomarkering platforms typifying IMC.

Hence, given the strong metastatic capacity of melanoma [5,7,34], we herein deeply mapped the proteomic landscape of WM115, human, primary (skin) melanoma cells and systemically compared it to the respective one derived from WM266-4 metastatic melanoma cells of the same patient [35]. Importantly, we unveiled novel and druggable metastatic biomarkering (systemic) signatures such as hybrid epithelial-to-mesenchymal transition (EMT)/mesenchymal-to-epithelial transition (MET) [31,33,36,37] and neural crest stem cell (NCSC) [37,38,39,40,41,42,43,44,45] oncogenic programs for mutant (e.g., V600D) BRAF-dependent human cutaneous melanomagenesis.

## 2. Materials and Methods

### 2.1. Antibodies, Drugs, and Chemicals

Rabbit monoclonal antibodies raised against (alphabetically ordered) ATF4, ATG7, cleaved (activated) Caspase-3^(Asp175)^ (a-Caspase-3), DR5, HIF1α, Keratin-5, LC3B, LOX, LOXL2, N-Cadherin, p63α, PDGFRβ, p-GSK3β-Ser^9^ (p-GSK3β), p-H2AX-Ser^139^ (p-H2AX), p-S6-Ser^235/236^ (p-S6), p-SMAD2-Ser^465/467^ (p-SMAD2), SLUG, SOX2, SOX9, SOX10, Vimentin, YAP, ZEB1, and ZEB2 were purchased from Cell Signaling Technology Inc. (Danvers, MA, USA). Rabbit polyclonal antibodies recognizing (alphabetically ordered) ATG12, β-Catenin, E-Cadherin, p-AKT-Ser^473^ (p-AKT), Pan-Actin, PCM1, p-p38-Thr^180^/Tyr^182^ MAPK (p-p38), p-p44/42-Thr^202^/Tyr^204^ MAPK (p-ERK1/2), p-p53-Ser^15^ (p-p53^Ser15^), p-p53-Ser^37^ (p-p53^Ser37^), p-SAPK/JNK-Thr^183^/Tyr^185^ (p-JNK), and p-STAT3-Tyr^705^ (p-STAT3) were obtained from Cell Signaling Technology Inc. (Danvers, MA, USA), while the respective ones against MCT1 and MCT4 were provided by Merck Millipore (Burlington, MA, USA). The rabbit polyclonal antibody raised against PRRX1 was purchased from Sigma-Aldrich (St. Louis, MO, USA). Mouse monoclonal antibodies against (alphabetically ordered) β-Tubulin, CHOP, Keratin-8/18, and TRA1-60(S) were supplied by Cell Signaling Technology Inc. (Danvers, MA, USA). The (goat) anti-rabbit IgG-Alexa Fluor-488 and anti-mouse IgG-Alexa Fluor-546 secondary antibodies used in immunofluorescence were provided by Invitrogen/Thermo Fisher Scientific (Waltham, MA, USA). The anti-rabbit and anti-mouse IgG-HRP secondary antibodies used in western blotting were supplied by Sigma-Aldrich (St. Louis, MO, USA), while the ECL (Enhanced ChemiLuminescence) western blotting reagents were purchased from GE Healthcare (Chicago, IL, USA). The rhodamine-phalloidin reagent (F-Actin probe) was obtained from Invitrogen/Thermo Fisher Scientific (Waltham, MA, USA), while the LY-364947 (TGF-β signaling) inhibitor was provided by Cell Signaling Technology Inc. (Danvers, MA, USA). Cisplatin was obtained from Ebewe Pharma GmbH Nfg. KG (Unterach am Attersee, Austria), Farmorubicin (epirubicin) was provided by Pfizer (New York, NY, USA), Pataxel (paclitaxel) was supplied by Vianex SA (Attica, Greece), Sorafenib was purchased from Cell Signaling Technology Inc. (Danvers, MA, USA), and Velbe (vinblastine) was supplied by Titolare AIC (Milan, Italy) (alphabetical order of drugs). All other chemicals were obtained from Sigma-Aldrich (St. Louis, MO, USA) and AppliChem GmbH (Darmstadt, Germany).

### 2.2. Cell Lines—Culture Conditions

The WM115 human melanoma cell line was derived from a primary cutaneous (right leg) tumor (VGP: vertical growth phase), whereas the WM266-4 melanoma cells have originated from individual lymph-node metastases (removed nine months after initial surgery of the primary lesion) of the same female patient [46,47]. Both human primary tumor (WM115) and metastatic (WM266-4) cutaneous melanoma cell lines feature the oncogenic mutation V600D (Val-600-Asp) at codon 600 in the *BRAF* gene. They were purchased from ECACC/Sigma-Aldrich (Munich, Germany), and cultured in 1× DMEM (Dulbecco’s modified Eagle’s medium) growth medium, supplemented with 10% FBS (fetal bovine serum), 2 mM L-glutamine, 1 mM sodium pyruvate, 50 mM sodium bicarbonate, 1× NEAA (non-essential amino acids), 100 U/mL penicillin, and 100 μg/mL streptomycin, at 37 °C, 5% CO_2_ and ≥95% humidity.

For high-scale and deep proteomics analysis, massive cultures of WM115 (primary tumor) cutaneous melanoma cells were harvested (on ice) via mild scrapping, and after three washes with (ice-cold) 0.9% NaCl, they were centrifuged (at +4 °C) for 10 min at 750× *g*. Supernatants were aspirated and cell pellets were stored at −20 °C for further proteomics processing. WM115 (primary) (this study) and WM266-4 (metastatic) [35] melanoma cells were treated and processed as similarly as possible to each other regarding culturing, harvesting, storage, and proteomics protocols, respectively applied. All cell culture media and related reagents were provided by Merck Millipore/Biochrom AG (Merck KGaA, Darmstadt, Germany).

### 2.3. Protein Extraction—Tryptic Peptide Generation

Technical protocols were employed as previously described [35,48]. Briefly, cell pellets derived from ~10^7^ cultured cells were suspended in lysis buffer containing 1.5 M Tris-HCl (pH 7.6), 3.5 M urea, 0.1 M SDS, and 3.2 mM DTE, and they were disrupted by tip sonication. Lysates were centrifuged at 13,000 × *g* for 20 min and total protein concentration was measured in each supernatant using the Bradford assay.

Protein lysates (~150 μg) were reduced and alkylated via treatment with 0.1 mM DTE in Tris-HCl (pH 6.8) at 56 °C for 30 min. Proteins were alkylated by the addition of 0.05 mM iodoacetamide at room temperature for 30 min in the dark. Samples were digested by trypsin (Roche/F. Hoffmann-La Roche Ltd., Basel, Switzerland) at a protein-to-trypsin ratio of 100:1, and trypsinization was terminated by the addition of 5% acetic acid. Peptide-containing solutions were vacuum-dried for 60 min, and powder preparations were reconstituted in 0.1% formic acid, for nano liquid chromatography-tandem mass spectrometry (LC-MS/MS) analysis.

### 2.4. LC-MS/MS—Data Analysis

Protocols for LC-MS/MS analysis were performed as previously described [35,48,49]. Briefly, each tryptic peptide mixture was separated in a linear gradient of 2–30% solution containing 99.9% acetonitrile and 0.1% formic acid, at a flow rate of 300 nL/min, on a C-18 column (75 μm × 50 cm, 100 Å, 2 μm bead-packed Acclaim Pepmap RSLC; Thermo Fischer Scientific, Waltham, MA, USA). An Ultimate-3000 System (Dionex/Thermo Fisher Scientific, Bremen, Germany) was coupled (on-line) to an Orbitrap Elite Instrument and mass spectra were collected in data-dependent acquisition mode using XCalibur^TM^ v.2.2 SP1.48 software (Thermo Fisher Scientific, Bremen, Germany). Full-scan data were obtained in the 300–2000 *m*/*z* range with a 60,000 resolution value and 100 milli-second maximum injection time. Data-dependent MS/MS for the 20 most intense ions per survey scan was performed, with HCD (higher-energy collision dissociation) fragmentation on the Orbitrap at a collision energy of 36 NSE% and a resolving power of 15,000. Fragments were analyzed on the Orbitrap, while the MS/MS spectra were acquired with a maximum injection time of 120 ms and a resolving power of 15,000. All measurements were carried out using *m*/*z* 445.120025 as the lock mass and dynamic exclusion was engaged within 45 s to prevent repetitive selection of the same peptide.

Raw data were processed via engagement of Proteome Discoverer (Thermo Fisher Scientific, Bremen, Germany), while protein identification was achieved by employment of the *Homo sapiens* proteome of reference derived from the UNIPROT database using the Sequest-HT v.28.0 algorithm (Thermo Fisher Scientific, Bremen, Germany). Search parameters were chosen as following: (a) two maximum missed cleavages for Trypsin; (b) oxidation of methionine as variable modification; (c) 0.05 ppm fragment ion tolerance; and (d) 10 ppm peptide mass tolerance. PSMs (peptide spectral matches) were validated using a percolator based on *q* values at 1% FDR (false discovery rate). Six amino acid residues were chosen as the minimum length of acceptable identified peptides.

### 2.5. Bioinformatics Platforms

Protein accession numbers retrieved from UNIPROT (Knowledgebase v.2.16) [50] were processed via engagement of the: (a) DAVID (Database for Annotation, Visualization and Integrated Discovery) [51,52,53]; (b) KEGG (Kyoto Encyclopedia of Genes and Genomes) [54,55]; (c) PANTHER (Protein Analysis Through Evolutionary Relationships) [56,57]; (d) dbEMT (Epithelial-Mesenchymal Transition Gene Database) [58,59]; (e) CMGene (Cancer Metastasis Gene Database) [60]; (f) LifeMap Discovery (Embryonic Development and Stem Cell Compendium) [61]; and (g) DepMapPortal (The Cancer Dependency Map at Broad Institute) [62,63,64,65] bioinformatics resources.

### 2.6. Immunofluorescence

Melanoma cells were seeded onto sticky *μ*-slide 8-well IBIDI plates (Ibidi GmbH, Martinsried, Germany), and the next day (~80% cell confluency) were fixed with 4% PFA (paraformaldehyde) in 1× PBS (phosphate-buffered saline), for 20 min at 37 °C and 5% CO_2_. After cell membrane permeabilization with 0.3% Triton X-100 in 1× PBS for 20 min at 37 °C and 5% CO_2_, slides were blocked with 1% BSA (bovine serum albumin), in 1× PBS (containing 0.3% Triton X-100) for 2 h at 37 °C and 5% CO_2_. Primary (polyclonal or monoclonal) antibodies (rabbit or mouse) were diluted in blocking buffer, according to the provider’s recommendations and slide incubation conditions were set as 60 min at room temperature, and (subsequently) 16 h at +4 °C. Secondary antibodies [IgG-Alexa Fluor-488 (anti-rabbit) and IgG-Alexa Fluor-546 (anti-mouse)] were used at 1:400 dilution for 2 h at room temperature in the dark. Cells were observed under a NIKON Digital Eclipse C1 CLSM (confocal laser scanning microscope) (Nikon Corporation, Tokyo, Japan) [66].

### 2.7. F-Actin Staining

F-Actin cytoskeleton was stained with rhodamine-conjugated phalloidin obtained from Invitrogen/Thermo Fisher Scientific (Wlatham, MA, USA). WM115 and WM266-4 melanoma cells were seeded onto *μ*-slide 8-well IBIDI plates (Ibidi GmbH, Martinsried, Germany) until they reached ~80% cell confluency. After the removal of growth medium, cells were washed with 1× PBS fixed for 20 min, in 1× PBS containing 4% formaldehyde, washed with 1× PBS, permeabilized for 20 min, in 1× PBS containing 0.3% Triton X-100 and washed again with 1× PBS. Next, cells were stained for 30 min with 100 nM rhodamine-phalloidin in the dark, washed with 1× PBS, covered with mounting medium, and immediately observed under a NIKON Digital Eclipse C1 CLSM (Nikon Corporation, Tokyo, Japan).

### 2.8. Lysosomal Staining

Lysosomal staining was performed using the LysoTracker-Red reagent (Invitrogen/Thermo Fisher Scientific, Waltham, MA, USA) in order to assess lysosomal acidification. WM115 and WM266-4 melanoma cells were seeded onto *μ*-slide 8-well IBIDI plates (Ibidi GmbH, Martinsried, Germany) until they reached ~80% cell confluency. Next, growth medium was removed, cells were washed three times with 1× PBS, and incubated for 30 min with 50 nM LysoTracker-Red at 37 °C in the dark. Subsequently, cells were washed again three times with 1× PBS, covered with mounting medium, and immediately observed under a NIKON Digital Eclipse C1 CLSM (Nikon Corporation, Tokyo, Japan).

### 2.9. Scratch Wound—Wound Healing Assay

The human melanoma cell lines WM115 (primary) and WM266-4 (metastatic) were cultured onto 100 mm Petri dishes and incubated for 24 h until 100% confluency (~2 × 10^6^ cells/dish). The next day, dish surfaces (cell monolayers) were mildly wounded (scratched) with a sterile 200 μL (yellow) tip and images were captured using a FE.2935 EUROMEX F range inverted microscope (Euromex Microscopen B.V., Arnhem, The Netherlands), a BMS 76458 microscope camera, and the ScopePhoto Software Program. Melanoma cells were treated with the LY-364947 inhibitor (100 μM) for up to 72 h at 37 °C and a 5% CO_2_ humidified environment. Control (untreated) and LY-364947-exposed melanoma cells were observed under an inverted microscope, and migration (motility) distances (wound-gap closures) were recorded (and quantified) at 0, 24, 48, and 72 h, post-treatment [67].

### 2.10. Cell Viability—MTT Assay

Melanoma cells were seeded onto 48-well plates and treated with different doses of each indicated drug (or the LY-364947 inhibitor) for 24 h, unless stated otherwise. Cells were incubated with MTT [3-(4,5-dimethylthiazol-2-yl)-2,5-diphenyltetrazolium bromide] solution for 4 h, and the formazan crystals produced were dissolved in pure isopropanol. Spectrophotometric absorbance was measured in a TECAN Infinite F50 absorbance microplate reader (Tecan Group Ltd., Männedorf, Switzerland) at 550 nm, using 630 nm as the wavelength of reference. Each cell viability (MTT) assay was repeated three times, using three wells per condition (e.g., absence or presence of drug/inhibitor) [66].

### 2.11. Western Blotting

Whole cell protein extracts (~50 μg) were separated in 12% SDS-PAGE gels and subsequently (electro-)transferred onto nitrocellulose membranes (Whatman-Schleicher & Schuell GmbH, Dassel, Germany). Membranes were blocked in 1× TBS-T (tris-buffered saline-Tween-20) containing 5% NFM (non-fat milk) for 2 h at room temperature. Primary antibodies were added at 1:1000 dilution for 2 h at room temperature and subsequently for 16 h at +4 °C. IgG-HRP (anti-rabbit, or anti-mouse) secondary antibodies were diluted 1:2000 and used for 2 h at room temperature, while the immuno-reacting protein bands were visualized by ECL reactions, following the manufacturer’s instructions. Pan-actin and β-tubulin served as proteins of reference (control) [66].

### 2.12. Molecular Modeling

Three dimensional (3D) predictions were generated by using the I-TASSER (Iterative Threading ASSEmbly Refinement) online server that has been designed for automated protein structure and function predictions [68,69]. Structural models of protein sequences were constructed from multiple threading alignments and iterative structural assembly simulations. Comparison of the produced models with other known protein structures can provide insights for the function of proteins being investigated [70].

The resulted molecular model of the KIAA0930-vimentin hybrid monomer protein was subjected to advanced docking bioinformatics processing, in order to examine its in silico ability to form homodimers via suitable employment of the automated protein docking server ClusPro [71,72,73,74]. ClusPro has proved able to yield energetically acceptable structural models in many rounds of the community-wide experiments called the CAPRI (Critical Assessment of PRedicted Interactions). CAPRI was designed to test protein docking algorithms in blind predictions of protein–protein complex structures [75] and to therefore identify the best and near-native conformations [73]. ClusPro consists of a search algorithm (Piper) that provides 1000 low-energy results to the clustering program. The 10 generated models form the central structure of the clusters that contain members within a 9 Å C-alpha RMSD radius. These are ranked initially according to the size of the cluster and subsequently according to the lowest energy due to the balanced scoring function of the program. Therefore, the top-ranked conformation of the complex is finally selected because of the good ranking performance of the program in the CAPRI challenge [75].

Images containing structural models were prepared by the PyMol Molecular Visualization System.

## 3. Results and Discussion

Both WM115 (primary) and WM266-4 (metastatic) melanoma cell lines were derived from the same patient and are considered as a powerful pre-clinical model system to thoroughly investigate the transition process from primary to metastatic melanoma. We have previously profiled the proteome of WM266-4 metastatic melanoma cells [35]. Thereby, to deeply map the WM115, primary melanoma cell proteome has reasonably emerged as an issue of major importance to the illumination of druggable mechanisms controlling cancer metastasis. To this direction, whole cell protein extracts derived from large-scale cultures of WM115 cells were subjected to a nano liquid chromatography-tandem mass spectrometry (nLC-MS/MS)-based proteomics analysis. This resulted in the identification, via UNIRPOT engagement, of 12,762 unique peptides and 3955 single proteins (Table S1). Comparison between the proteomic contents of WM115 (primary) (this study; Table S1) and WM266-4 (metastatic) [35] melanoma cells revealed 812 and 3538 proteins exclusively expressed in WM115 and WM266-4 cells, respectively (Tables S2 and S3) (see also Figure 10A). Intriguingly, the WM115 proteome (3955 single proteins) (Table S1) seems to contain ~40% numerically fewer components than the WM266-4 one (6681 single proteins) [35], thus indicating the metastasis-induced upregulation of gene expression and protein synthesis programs.

### 3.1. Functional Mapping of WM115 Proteomic Landscape Unveils Diverse Pathways Being Engaged in Signal Transduction, Drug Metabolism/Resistance, Cell Death, and Cytoskeleton Re-modeling

**Figure 1:**
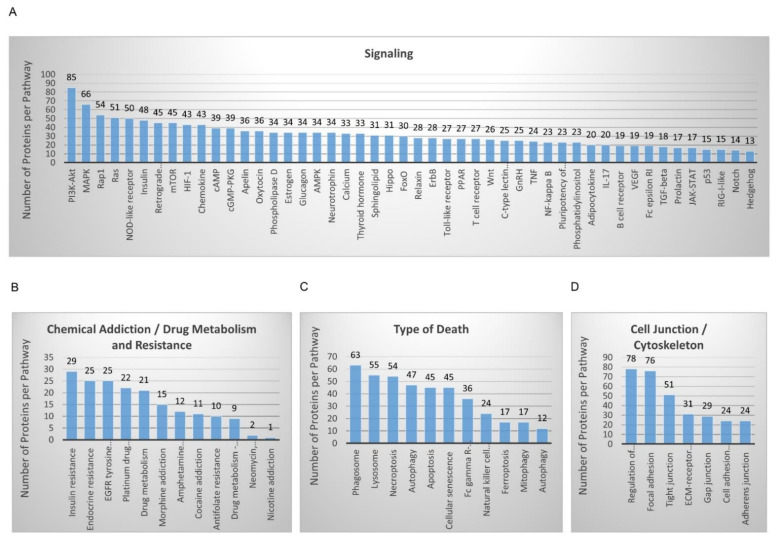
Dissection of WM115 deep proteome components in diverse functional networks. (**A**) “Signaling”. (**B**) “Chemical Addiction/Drug Metabolism and Resistance”. (**C**) “Type of Death”. (**D**) “Cell Junction/Cytoskeleton”. (**A**–**D**) KEGG was employed as the suitable bioinformatics tool for the analysis. The number of proteins in the identified sub-routines (pathways) are shown on the top of the respective bars. (**A**) “PI3K-AKT”, “MAPK”, “RAP1”, “RAS”, “NOD-like receptor”, “Insulin”, “Retrograde Endocannabinoid”, “mTOR”, “HIF-1”, “Chemokine”, “cAMP”, “cGMP-PKG”, “Apelin”, “Oxytocin”, “Phospholipase D”, “Estrogen”, “Glucagon”, “AMPK”, “Neurotrophin”, “Calcium”, “Thyroid hormone”, “Sphingolipid”, “HIPPO”, “FOXO”, “Relaxin”, “ERBB”, “TOLL-like receptor”, “PPAR”, “T cell receptor”, “WNT”, “C-type Lectin receptor”, “GnRH”, “TNF”, “NF-kappa B”, “Pluripotency of stem cells”, “Phosphatidylinositol”, “Adipocytokine”, “IL-17”, “B cell receptor”, “VEGF”, “Fc epsilon RI”, “TGF-beta”, “Prolactin”, “JAK-STAT”, “p53”, “RIG-I-like”, “NOTCH”, and “HEDGEHOG”. (**B**) “Insulin resistance”, “Endocrine resistance”, “EGFR tyrosine kinase inhibitor resistance”, “Platinum drug resistance”, “Drug metabolism”, “Morphine addiction”, “Amphetamine addiction”, “Cocaine addiction”, “Antifolate resistance”, “Drug metabolism—Cytochrome P450”, “Neomycin, Kanamycin and Gentamicin biosynthesis”, and “Nicotine addiction”. (**C**) “Phagosome”, “Lysosome”, “Necroptosis”, “Autophagy—animal—Homo sapiens (human)”, “Apoptosis”, “Cellular senescence”, “Fc gamma R-mediated phagocytosis”, “Natural killer cell-mediated cytotoxicity”, “Ferroptosis”, “Mitophagy”, and “Autophagy—other—Homo sapiens (human)”. (**D**) “Regulation of Actin cytoskeleton”, “Focal adhesion”, “Tight junction”, “ECM-receptor interaction”, “Gap junction”, “Cell Adhesion Molecules (CAMs)”, and “Adherens junction”. Due to their specific functional traits, certain proteins are classified in more than one pathway.

The KEGG-mediated categorization of WM115 proteomic content resulted in the construction of 48 “signaling” courses (Figure 1A), 12 “chemical addiction/drug metabolism and resistance” routes (Figure 1B), 11 “type of death” programs (Figure 1C), and seven “cell junction/cytoskeleton” processes (Figure 1D). Forty-eight “kinases” and nine “phosphatases” were specifically identified in WM115 (primary melanoma) cells (Table S2), while 200 “kinases” and 78 “phosphatases” were exclusively observed in WM266-4 (metastatic melanoma) cells (Table S3) (see also Figure 10G,H and Tables S20–S23) (GO_MF DAVID 6.8; [51,52,53]), thus indicating the metastasis-dependent elevation of phosphorylation/de-phosphorylation incidents (and dynamics) in advanced melanoma. Among the bioinformatically assembled pathways, “PI3K-AKT” (*n* = 85) and “MAPK” (*n* = 66) presented with the highest number of respective components (Figure 1A) with “Insulin Resistance” (*n* = 29), “Endocrine Resistance” (*n* = 25), “EGFR Tyrosine Kinase Inhibitor Resistance” (*n* = 25), and “Platinum Drug Resistance” (*n* = 22) being the most numerically enriched groups of the cluster (Figure 1B).

Interestingly, the WM115 proteomic catalogue contains the *MAPKAPK2*/MAPK2 [2.25 (MS: Mascot Score)], *MAP3K13*/M3K13 (0.00), and *MAP4K2*/M4K2 (0.00) kinases (Table S2) (see also Table S20), whereas the WM266-4 protein collection includes the *MAP3K7*/M3K7 (9.62), *MAP2K4*/MP2K4 (3.17), *MAP2K5*/MP2K5 (2.92), *MAPK12*/MK12 (2.13), *MAP2K6*/MP2K6 (2.09), *MAPKAPK3*/MAPK3 (1.75), *MAP3K3*/M3K3 (0.00), *MAP3K4*/M3K4 (0.00), *MAP3K10*/M3K10 (0.00), *MAP4K3*/M4K3 (0.00), *MAP4K5*/M4K5 (0.00), and *MAPKAPK5*/MAPK5 (0.00) kinases (Table S3) (see also Table S22), strongly suggesting the major importance of the MAPKKK/MAPKK/MAPK-specific signaling repertoire in controlling human melanoma metastasis. Similarly, the *PRKAA2*/AAPK2 (9.40) kinase (subunit) seems to be exclusively expressed in WM115 (Table S2) (see also Table S20), while the *PRKAA1*/AAPK1 (3.36) family member is solely detected in WM266-4 cells (Table S3) (see also Table S22), presumably pointing out the suppressive and supportive role of AAPK2 and AAPK1 (5′-AMP-Activated Protein Kinase Catalytic Subunit Alpha-2/1), respectively, in the transition from primary to metastatic melanoma. Since deletion of the gene *UBE2O*, whose product (UBE2O) (WM115, MS: 0.00, Table S1; WM266-4, MS: 23.43, [35]) specifically targets AAPK2 (AMPKα2) for ubiquit(in)ylation and degradation (thereby, promoting the mTOR-HIF1α axis activation), impairs tumor initiation, growth, and metastasis [76], WM266-4 cells may have undergone an UBE2O-mediated downregulation of AAPK2 to become metastatic. Nevertheless, transcriptional suppression of the gene encoding AAPK1 (AMPKα1) can promote breast cancer metastasis upon oncogene activation [77], thus dictating the dependence of AAPK1 to act as a metastasis inducer (e.g., in WM266-4) only in particular mutational settings (e.g., BRAF^V600D^) with strong melanomagenic capacities.

Remarkably, HIF1α (Hypoxia-Inducible Factor-1α) (0.00) is exclusively observed in the WM266-4 proteomic map (Table S3) (see also Table S24), supporting its critical role in metastatic melanomagenesis. Indeed, pharmacological targeting of HIF1α-dependent transcription machinery can reduce primary tumor growth and metastasis of uveal melanoma [78]. Furthermore, HIF1(α) is able to promote metastasis of pancreatic ductal adenocarcinoma by activating transcription of the Actin-bundling protein Fascin (FSCN1) [79]. Most importantly, both FSCN2 (0.00) and FSCN3 (0.00) family members are contained in the WM266-4, but not WM115, proteomic landscape (Tables S2 and S3), indicating their presumable transcriptional regulation by HIF1α, and their implication in the progression of melanoma to metastatic disease. Besides HIF1α, several other transcription factors are also subjected to differential expression between WM115 (primary) and WM266-4 (metastatic) melanoma cells. Although the cAMP-dependent transcription factor ATF2 (5.31) is identified solely in WM266-4 (Table S3), the ATF1 (0.00) and ATF6B (0.00) (super-)family members are recognized specifically in WM115 cells (Table S2) (see also Tables S24 and S25). Given that ATF2-derived peptides can induce inhibition of melanoma growth and metastasis [80], a new therapeutic window seems to open for the metastatic disease by targeting its oncogenic ATF2-mediated signaling. Similarly, several Forkhead Box (FOX) proteins present a cell type-specific expression profiling, with the FOXK1 (3.97), FOXH1 (0.00), FOXL1 (0.00), and FOXN1 (0.00) transcription factors exclusively identified in WM266-4 cells (Table S3) (see also Table S24). The previously recognized contribution of FOXK1 to the advancement of cancer to metastatic stage(s) [81,82,83] fosters the mechanistic and therapeutic association of FOXK1 elevated content(s) with metastases of melanoma(s) in the clinic.

The insulin-degrading enzyme (IDE) represents another protein that is differentially expressed between the two cell types herein examined. IDE (5.82) is solely identified in WM266-4 metastatic melanoma cells (Table S3), and according to its vital role in insulin degradation and clearance [84], it may serve as a metastatic driver of human melanoma by suppressing the presumable ability of insulin to inhibit cell invasion and metastasis. Interestingly, leptin and insulin can decrease the invasiveness of colon cancer cells, with insulin (or/and leptin) resistance likely increasing the metastasis risk [85]. Since IDE was not recognized in the WM115 proteomic map (Table S1), primary melanoma cells (e.g., WM115) could activate an IDE-independent, distinct, pathway to achieve “insulin resistance” (*n* = 29) (Figure 1B) required for their future metastatic fate. The *IGFBP3*/IBP3 (16.79) and *IGFBP5*/IBP5 (3.51) members of the insulin-like growth factor-binding protein family are also presented with a WM266-4-specific expression patterning (Table S3), likely deregulating the bioavailability and signaling power of their cognate insulin-like growth factors (IGFs). IBP3 upregulation has been tightly associated with brain metastasis in lung adenocarcinoma [86], tumor metastasis in nasopharyngeal carcinoma [87], and TGF-β-dependent colorectal cancer cell migration and invasion [88]. Hence, it seems that IBP3 may act as principal metastatic driver in BRAF^V600D^ melanoma cell environments, with its targeted drugging presumably offering new opportunities for successful management of the advanced disease.

Surprisingly, WM266-4 but not WM115 cells were shown to carry high levels of the *DAP*/DAP1 proteomic component (96.65) (Table S3), a small, proline-rich, cytoplasmic protein, which belongs to the death-associated protein family [89,90]. Activated (de-phosphorylated) DAP1 negatively regulates autophagy, while its elevated expression increases the risk of lymph-node metastasis in squamous cell carcinoma of the oral cavity [91,92,93]. Thereby, DAP1 could be endowed with a property to promote melanoma advancement from primary (e.g., WM115) to the metastatic (e.g., WM266-4) stage by downregulating the autophagic machinery whose functionality may differ between WM266-4 [35] and WM115 cells (Figure 1C; *n* = 47) (see also Figure 8G). Given that DAP1 has been previously suggested to act as a positive mediator of IFN-γ-induced programmed cell death [90], an IFN-γ-based, novel, drug-cocktail scheme might prove beneficial for metastatic melanoma patients in the clinic. Intriguingly, PGAM5 (10.39), a mitochondrial serine/threonine-protein phosphatase that functions at the convergence point of several necrotic/necroptotic death pathways [94], is also presented with a WM266-4-specific pattern of expression (Table S3), therefore indicating a surprising role of necrosis/necroptosis in melanoma metastasis. Indeed, it has been previously reported that necroptosis of cancer cells can cause tumor necrosis and can promote tumor metastasis in certain oncogenic settings [95]. It is likely, and really astonishing, that the major cell death programs such as apoptosis, autophagy, and necroptosis (and ferroptosis) can critically contribute to the metastatic process of melanoma [35] (Figure 1C) and other tumors through yet undiscovered, novel, cross-talks among tumor cell sub-populations and their micro-environments.

Given the differential adhesion properties usually acquired by primary versus metastatic tumor cells, WM115 and WM266-4 proteomic maps were thoroughly examined for their “adhesion”-related contents (Figure 1D) [35]. The BCAM (6.55), CADM3 (2.74), NCAM1 (1.77), and EPCAM (0.00) proteins were exclusively identified in WM115 primary melanoma cells (Table S2), while the L1CAM (22.25), *CHL1*/NCHL1 (10.76), NRCAM (2.34), JAM3 (1.71), *CEACAM19*/CEA19 (0.00), and ICAM5 (0.00) proteomic components were solely observed in WM266-4 metastatic melanoma cells (Table S3). Since L1CAM-positive cells in human colorectal cancer have been shown to carry a metastasis-initiating capacity [96,97], L1CAM can be considered as major metastatic driver of BRAF^V600D^ human melanoma. Indeed, L1CAM overexpression has been previously associated with metastasis in cutaneous malignant melanoma [98]. Hence, by taking into account the powerful technology of L1CAM-specific therapeutic antibodies in pre-clinical models [99,100], an anti-L1CAM humanized monoclonal antibody-based (immuno-)therapy, combined with targeted chemotherapy (e.g., BRAF^V600D^ inhibitors), may become a helpful tool for the clinical management of BRAF^V600D+^/L1CAM^+^ metastatic melanoma tumors.

Since melanin has been previously reported to compromise the chemo-, radio-, and immuno-therapy efficacies in metastatic melanomas [101,102,103,104], we next investigated the proteomic content that critically controls melanogenesis in WM115 and WM266-4 melanoma cells. In contrast to the WM266-4 proteome (Table S3, [35]), WM115 (in our cell growth setting) was presented to lack the MITF, *TYR*/TYRO, TYRP1, and *DCT*/TYRP2 proteins (Table S1) that serve as major determinants of melanogenesis [7,105,106,107,108], thus indicating the inability of WM115 cells to initiate a melanogenesis-specific program, further confirming their unpigmented character described in previous reports [109,110]. It may be that this rather amelanotic phenotype likely renders primary melanoma (e.g., WM115) cells more sensitive and responsive to certain therapeutic schemes compared to metastatic melanoma (e.g., WM266-4) cells in the clinic.

### 3.2. WM115 and WM266-4 Human Melanoma Cells Are Subjected to Hybrid Epithelial-to-Mesenchymal Transition (EMT)/Mesenchymal-to-Epithelial Transition (MET) Programs

**Figure 2:**
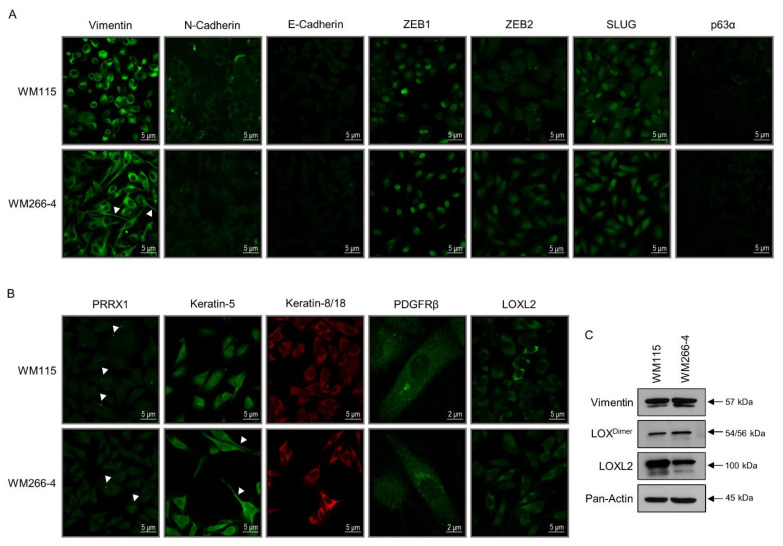
Engagement of hybrid EMT/MET programs during BRAF^V600D^-dependent melanomagenesis. (**A**,**B**) Immunofluorescence profiles of epithelial (MET) and mesenchymal (EMT) program protein markers in WM115 (primary) and WM266-4 (metastatic) melanoma cells. (**C**) Western blotting-derived patterns of protein expression being critically implicated in the acquisition of epithelial or mesenchymal (molecular/phenotypic) traits. (**A**) “Vimentin” (M), “N-Cadherin” (M), “E-Cadherin” (E), “ZEB1” (M), “ZEB2” (M), “SLUG” (M) and “p63α” (E). (**B**) “PRRX1” (M), “Keratin-5” (E), “Keratin-8/18” (E/M), “PDGFRβ” (E/M), and “LOXL2” (M). (**C**) “Vimentin” (M), “LOX” (M), “LOXL2” (M), and “Pan-Actin” (protein of reference). E: Epithelial (MET) marker. M: Mesenchymal (EMT) marker. Arrowheads: (**A**) “Vimentin”-positive Invadopodia and (**B**) “PRRX1”-positive globular “specks”; “Keratin-5”-positive Invadopodia. (**A**,**B**) Scale bars: 2 or 5 μm. (**C**) Molecular weights of identified proteins (major bands) are denoted by numbers at the right side of each respective panel. Protein quantification values (in bar-chart format) are shown in Figure S1.

EMT is a reversible cellular process during which epithelial cells acquire mesenchymal characteristics and downregulate their epithelial features. Cells display fibroblast-like morphology and architecture as well as increased migratory activity and invasion capacity. Most importantly, EMT has been tightly associated with tumor initiation and progression, tumor stemness, metastasis, and resistance to therapy [36,37,38,39,40,41,111,112]. Thereby, we examined the expression profiles of several proteins that serve as bona fide epithelial (e.g., *CDH1*/cadherin-1/E-cadherin) or mesenchymal (e.g., *VIM*/VIME/vimentin) markers [37,38,39,40,41,111,112,113] to investigate the engagement of EMT or/and MET programs in primary (WM115) and metastatic (WM266-4) melanoma development. Strikingly, vimentin is presented as the most abundantly expressed protein in both WM115 (1,703.82) (Table S1) and WM266-4 (1638.22) [35] proteomic maps (see also Table S10), thus clearly indicating that both WM115 (primary) and WM266-4 (metastatic) melanoma cells have undergone an EMT process. The strong vimentin’s immunofluorescence patterns (Figure 2A) and western blotting profiles (Figure 2C) undoubtedly confirm the proteomic landscape (MS-derived) quantifications for both cell types, and demonstrate the acquisition of a vimentin-dependent mesenchymal program in primary and metastatic melanoma environments. Lack of E-cadherin detection (Table S1 [35] and Figure 2A) indicates the complete suppression of an E-cadherin-specific program controlling epithelial differentiation in WM115 and WM266-4 cells. Similarly, neither in proteomic maps (Table S1, [35]) nor immunofluorescence profiles (Figure 2A), the *TP63*/*TRP63*/tumor protein 63 (p63α) epithelial transcription factor that typifies EMT transition states [38,39,113] could be identified, thus suggesting the p63α-independent transition from primary (WM115) to metastatic (WM266-4) melanoma.

In contrast to E-cadherin, the other member of the family, *CDH2*/CADH2/N-cadherin, which serves as a major mesenchymal marker [37,38,39,40,41,113], exhibited positive staining in a significant number of WM115, but not WM266-4 cells (Figure 2A). However, N-cadherin could be recognized in both WM115 (27.00) (Table S1) and WM266-4 (19.84) [35] proteomes (see also Table S10), thereby dictating the metastasis-induced antigenicity re-modeling (e.g., via post-translational modifications, or/and mRNA splice variants production) of N-cadherin in human melanoma. A rather inverse immunofluorescence patterning was observed for the ZEB2 transcription factor, with its nuclear expression levels being notably higher in WM266-4 than WM115 melanoma cells (Figure 2A). The transcription factors ZEB1, ZEB2, *SNAI1*/SNAIL, and *SNAI2*/SLUG act as principal regulators of EMT through repression of the epithelial state and induction of the mesenchymal state. For example, *ZEB* and *SNAI* gene family products can directly bind onto the *CDH1* promoter, causing its cognate gene silencing [36,37,39,40,41,45,111,112,114,115]. Remarkably, the strong nuclear immunofluorescence signal of ZEB1 in both WM115 (primary) and WM266-4 (metastatic) melanoma cells (Figure 2A) is mechanistically associated with their inability to transcriptionally activate the *CDH1* gene (Figure 2A). Despite its absence from both proteomic collections (Table S1 [35]), SLUG protein presented a clear and intense nuclear compartmentalization only in WM115 cells (Figure 2A), showing a cell type-specific and rather opposite distribution pattern compared to the ZEB2 protein. It seems that primary (WM115) and metastatic (WM266-4) melanoma cells employ different combinations (and intracellular quantities/topologies) of transcription factors to direct their respective EMT programs.

Strikingly, different EMT tumor transition states have been molecularly characterized. They seem to carry distinct metastatic potentials, with the *TP63*/*TRP63*, *ZEB1/2*, *SNAI1/2*, and *PRRX1* gene products differentially specifying the: (a) epithelial EMT, (b) early-hybrid EMT, (c) late-hybrid EMT, and (d) full EMT state(s) [38,39,113]. The PRRX1 transcription factor is an EMT inducer, conferring migratory and invasive capacities, while its loss is required for cancer cell metastasis in vivo [112,116]. PRRX1 isoform switching from 1b to 1a stimulates metastatic colonization [117], with PRRX1b promoting tumor invasion, de-differentiation, and EMT [117,118], and PRRX1a supporting metastatic outgrowth, tumor differentiation, and MET [117]. Surprisingly, the PRRX1 immunofluorescence profiling in WM115 and WM266-4 (0.00) (Table S3) cells revealed a mainly cytoplasmic compartmentalization of the protein with 1–2 strong globular “specks” of diverse sizes being recognized per signal-positive melanoma cell (Figure 2B). Intriguingly, infrequent incidents of more than two “specks” per immunoreacting cell were detected. Nuclear localization was observed only in a few WM115 cells, whereas numerous cells of both types (primary and metastatic) exhibited the cytoplasmic “specked” patterning. Taken together, it may be the nuclear exclusion of PRRX1 (one, or more of its isoforms: splice variant products) that compels primary (WM115) and metastatic (WM266-4) melanoma cells to acquire a MET program.

Next, to examine if melanoma cells are, indeed, being subjected to MET (“reverse” EMT) or MET-like programs, the WM115 (primary) and WM266-4 (metastatic) melanoma cells were analyzed for the expression of MET-typifying markers. Even though WM115 and WM266-4 cells proved negative for E-cadherin immunostaining (Figure 2A), they showed positive immunofluorescence profiling for *KRT5*/K2C5/keratin-5 (WM266-4, MS: 11.87; Table S3 [35]) and *KRT8/18*/K2C8/K1C18/keratin-8/18 proteins, with some WM266-4 cells featuring comparatively stronger signals (Figure 2B). Since keratin-5 serves as a typical epithelial component of EMT [113,114] and significantly contributes to epithelial EMT, early-hybrid EMT, and late-hybrid EMT [38], both WM115 (primary) and WM266-4 (metastatic) melanoma cells must have undergone a keratin-5-dependent re-programming toward MET. Similarly, keratin-8 characterizes the epithelial and hybrid epithelial/mesenchymal (EMT/MET) states [114], thus dictating the acquisition of hybrid EMT/MET programs from both primary and metastatic melanoma cells. Notably, L1CAM, an epithelial adhesion molecule (EAM) [113], can be identified in the WM266-4 (22.25) (Table S3, [35]), but not WM115 (Table S1) proteomic catalogue, strongly suggesting the metastasis-induced activation of an L1CAM-specific MET program in human melanoma. On the other hand, the WM115 proteomic map contains the EPCAM (0.00) (Tables S1 and S2) protein, which serves as an EAM and epithelial surface marker (ESM) [38], thereby indicating that primary melanoma (e.g., WM115) cells can engage an EPCAM-dependent MET program.

To more accurately determine the hybrid EMT/MET states (early or late [38]), WM115 and WM266-4 cells were processed for immunofluorescence detection of PDGFRβ, a signaling membrane receptor that typifies the late-hybrid EMT state [38]. Remarkably, many WM115 (primary) and WM266-4 (metastatic) melanoma cells proved positive for PDGFRβ immunostaining, carrying diverse-sized cytoplasmic “specks” (Figure 2B). Hence, it seems that distinct WM115 and WM266-4 cell sub-populations have entered a PDGFRβ-mediated late-hybrid EMT program. Different tumor (e.g., melanoma) cell sub-populations may be associated with different EMT stages, ranging from completely epithelial (full MET) to completely mesenchymal (full EMT) ones, and passing through intermediate hybrid (EMT/MET) states that confer distinct cell plasticity, invasiveness, and metastatic potential [39,113]. Hybrid EMT/MET phenotypes likely provide navigating tumor (e.g., melanoma) cells with survival advantages in adverse micro-environments such as blood and lymphatic vessels, and secondary tumor sites [36]. Mesenchymal properties are required for the intravasation of cells from primary tumor (e.g., melanoma) and their survival in blood circulation, whereas epithelial traits seem to be indispensable for metastatic colonization at distant sites [33,36,114]. Interestingly, spontaneous metastasis of primary tumors has been recently associated with MET program activation [119], although MET-independent mechanisms may significantly contribute to the higher metastatic capacities of hybrid EMT/MET-undergoing cell sub-populations [39,113]. Epithelial (MET) and mesenchymal (EMT) cell traits probably need to be co-expressed within individual tumor (e.g., melanoma) cells, rather than in exchanging signal distinct (either EMT or MET) cells for efficient tumorigenesis (e.g., melanomagenesis) including metastasis to unfold. Although both WM115 (primary) and WM266-4 (metastatic) BRAF^V600D^ melanoma cells have activated hybrid EMT/MET programs, WM266-4 cells seem to have acquired a rather stronger epithelial (MET) character, thus revealing their higher propensity to melanoma outgrowth and distant metastasis.

Strong evidence has emerged for the critical role(s) of lysyl oxidase (LOX) family members in promoting metastasis [120] with LOX, an extracellular matrix-modifying enzyme, having been proved essential for hypoxia-induced metastasis [121,122]. Thereby, via western blotting, LOX cellular contents were examined in both WM115 and WM266-4 cells. Interestingly, both cell types presented similar LOX (dimer) expression levels (Figure 2C), indicating the proclivity of primary melanoma cell sub-populations toward LOX-mediated metastasis. Given that the lysyl oxidase homolog 2 (LOXL2) upregulation seems to cause tumor progression and metastasis through re-modeling of the tumor micro-environment [120,123], LOXL2 was investigated by immunofluorescence (Figure 2B) and western blotting (Figure 2C) protocols in the two cell types. Strong expression was detected in either WM115 (primary) (2.33; Tables S1 and S2) or WM266-4 (metastatic) melanoma cells, with WM115 carrying higher (cytoplasmic) protein levels compared to the WM266-4 respective ones (Figure 2B,C). It seems that, similar to LOX, LOXL2 may also foster primary (e.g., WM115) melanoma cells to precociously gain metastatic traits, phenotypically resembling melanoma “mature” metastases (e.g., WM266-4). Importantly, LOX and LOXL2 enzymes have been reported to mediate a HIF1-dependent EMT program induction in response to hypoxia [120,124]. Taken together, primary (e.g., WM115) and metastatic (e.g., WM266-4) melanoma cells likely engage LOX family members to activate EMT repertoires and trigger time-specific metastases.

### 3.3. TGF-β Signaling Controls WM115 and WM266-4 Cell Motility In Vitro

**Figure 3:**
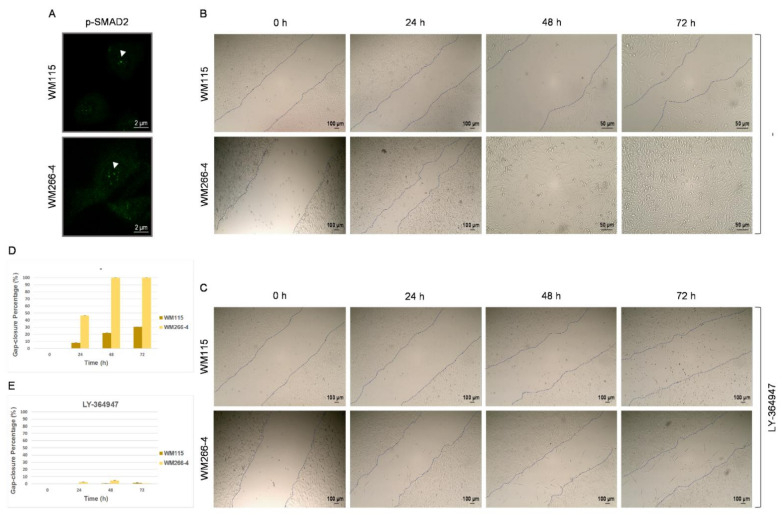
In vitro migration of WM115 and WM266-4 human melanoma cells depends on TGF-β/SMAD2 signaling axis. (**A**) Immunofluorescence patterns of (activated) “p-SMAD2” protein in WM115 (primary) and WM266-4 (metastatic) melanoma cells. “p”: Phosphorylation. Arrowheads: “p-SMAD2”-positive nuclear “specks”. Scale bars: 2 μm. (**B**,**C**) Light micrographs of wound healing (scratch wound) assays, examining the motility/migration capacities of WM115 and WM266-4 melanoma cells, in the absence (**B**) or presence (**C**) of the TGF-β signaling specific inhibitor LY-364947 (100 μM), for a time-frame of 0–72 h. (**B**) Scale bars: 50 or 100 μm. (**C**) Scale bars: 100 μm. Dash lines denote the melanoma cell migration borders. (**D**,**E**) Quantification, in bar-chart format, of in vitro motility/migration activities, as demonstrated by gap-closure percentages (%) (mean values) in wound healing assays (**B**,**C**) of WM115 (primary) and WM266-4 (metastatic) human melanoma cells in the absence (**B**,**D**) or presence (**C**,**E**) of the LY-364947 inhibitor (0–72 h).

The TGF-β signaling pathway leads to activation of EMT program(s) through several distinct mechanisms, with the TGF-β-induced SMAD complexes transcriptionally turning on mesenchymal genes (e.g., *VIM*) or turning off epithelial genes such as *CDH1*, via, among others, upregulation of the EMT-specific transcription factors SNAIL, SLUG, and ZEB1. Thereafter, critical EMT regulators (e.g., SNAIL) can activate an autocrine TGF-β-dependent signaling, creating a positive feedback loop that helps cells to maintain their EMT repertoire(s) once established [37,41,45,111,112,125,126,127,128]. Hence, we next examined the expression profile of phosphorylated SMAD2 (p-SMAD2) transcription factor, a major mediator of TGF-β signaling [37,125,128,129], in WM115 and WM266-4 cells (Figure 3A). Both primary (WM115) and metastatic (WM266-4) melanoma cells presented with similar immunofluorescence patterns, being mainly featured by few nuclear “specks” (of diverse size and shape) per positive (immunoreacting) cell, thus indicating the TGF-β/SMAD(2) axis functional engagement in both primary and metastatic melanoma environments. Altogether, an autocrine TGF-β signaling route and a p-SMAD2-dependent induction of EMT program(s) are strongly suggested to successfully operate both in primary and metastatic forms of human BRAF^V600D^ melanoma.

To investigate the role(s) of TGF-β signaling in melanoma cell motility and migration, two fundamental EMT features [36,37,38,45,111,112,128], a wound healing assay, in the presence or absence of LY-364947 that serves as a specific TGF-β signaling inhibitor [126,130], was suitably conducted in vitro. In contrast to WM115, WM266-4 cells proved able to carry strong motility capacities with a closing gap completion time between 24 and 48 h (Figure 3B,D). The significantly lower migration speed of WM115 cells (Figure 3B,D) likely reflects their primary tumor (e.g., melanoma) character, while the comparatively higher speed of WM266-4 cells to fill the gap in vitro (Figure 3B,D) proclaims their strong migratory, invasive, and metastatic abilities.

Remarkably, exposure of WM115 (primary) and WM266-4 (metastatic) melanoma cells to the LY-364947 inhibitor compelled both cell types to completely cease migration toward the artificial gap-closure (Figure 3C,E), thus evidencing the strong dependence of primary and metastatic melanoma cell motility/migration processes by TGF-β (autocrine) signaling in vitro. Of note, many cell death incidents could be specifically recognized in the LY-364947-treated (72 h) WM115 cell (sub-)populations, whereas several large-sized and shape-elongated cells were rather exclusively observed inside the respective gaps of WM266-4 culture monolayers in response to LY-364947 administration for 72 h (Figure 3C). Clonal isolation, molecular characterization, and chemical targeting of metastatic melanoma cell escapers generated during LY-364947 treatment may open new windows for the successful management of advanced disease.

### 3.4. WM115 and WM266-4 Melanoma Cells Feature Different Neural Crest-Like Stemness Signatures

**Figure 4:**
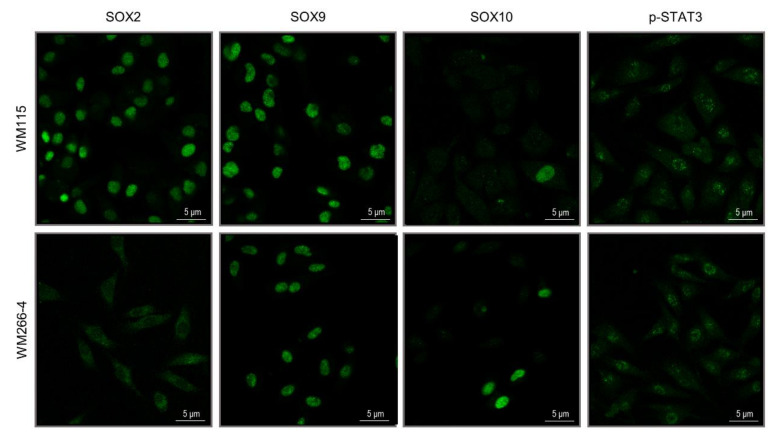
IMC-dependent NC-like stemness signatures of BRAF^V600D^-positive melanoma cells. Immunofluorescence phenotyping of “SOX2”, “SOX9”, “SOX10”, and (activated/phosphorylated) “p-STAT3” transcription factors in WM115 (primary) and WM266-4 (metastatic) human BRAF^V600D^ melanoma cells. “p”: Phosphorylation. Scale bars: 5 μm.

A mechanistic association between EMT programs/states and acquisition of stem cell-like properties has been previously described. Exposure to TGF-β or forced expression of EMT-inducing transcription factors can give rise to cell (sub-)populations that present stem cell or stem cell-like properties. When carcinomas are involved, these stem-like cells are usually specified as cancer stem cells (CSCs) [37,38,39,40,41,42,43,44,45]. Since members of the SOX protein family serve as versatile regulators of stem- and progenitor-cell fates [131,132,133,134,135], we next investigated the expression patterns of SOX2, SOX9, and SOX10 transcription factors in WM115 (primary) and WM266-4 (metastatic) melanoma cells via an immunofluorescence protocol employment (Figure 4). SOX2 proved to be highly accumulated in all WM115 cell nuclei, while its detection could hardly be recognized in WM266-4 cells. SOX9 was strongly expressed in all WM115 and WM266-4 melanoma cells, with the WM115-specific nuclear immunostaining being rather notably enhanced compared to the WM266-4 respective one. Remarkably, SOX10 expression could be detected only in a few cells, thus unveiling the SOX10-directed cell (sub-)population heterogeneity, with the number of SOX10-positive nuclei in WM266-4 (70.42; [35]) being comparatively higher than the one in WM115 (9.66; Table S1) cells (Figure 4) (see also Table S10).

Furthermore, given the pivotal role(s) of cytokine(s)/JAK(s)/STAT3 signaling axis(es) in the acquisition of CSC phenotypes [136,137,138], the activated (phosphorylated) STAT3 (p-STAT3) nuclear content in both WM115 and WM266-4 cells was also examined. The obtained immunofluorescence profiles revealed relatively weak, but detectable, p-STAT3-specific punctuated signals in many WM115 and WM266-4 cell nuclei (Figure 4), indicating cytokine(s) (e.g., IL-6)-driven, cell-autonomous, signaling toward development of an activated STAT3-mediated stemness in BRAF^V600D^ primary and metastatic melanoma cells.

Melanocytes derive from neural crest stem cells (NCSCs) that disseminate into the embryo to form an array of diverse cell lineages [139]. Neural crest (NC) specification and migration modules contain critical SOX family members such as SOX9, SOX10 (Figure 4), and SOX5 (WM266-4, MS: 3.07; Table S3, [35]), together with the *TFAP2(A)*/AP2(A) (WM115, MS: 0.00; WM266-4, MS: 2.34; Table S1, [35]) and RXRG (WM266-4, MS: 5.70; Table S3 [35]) program regulators [140]. Taken together, it seems that WM115 (primary) and WM266-4 (metastatic) melanoma cells carry NC-like stemness properties that can be typified, among others, by distinct SOX protein expression profiles. Notably, since *SAMMSON*, a long non-coding RNA (lncRNA) gene, is transcriptionally regulated by SOX10 in melanoma cells [141], while its product can orchestrate the self-renewal of liver CSCs [142], it may act as a major determinant of IMC in BRAF^V600D^ melanoma environments, rendering the SOX10-*SAMMSON* axis a promising target for metastatic melanoma therapy in the clinic.

### 3.5. Cytoskeleton Architecture Re-Modeling of Melanoma Cells during the IMC Process

**Figure 5:**
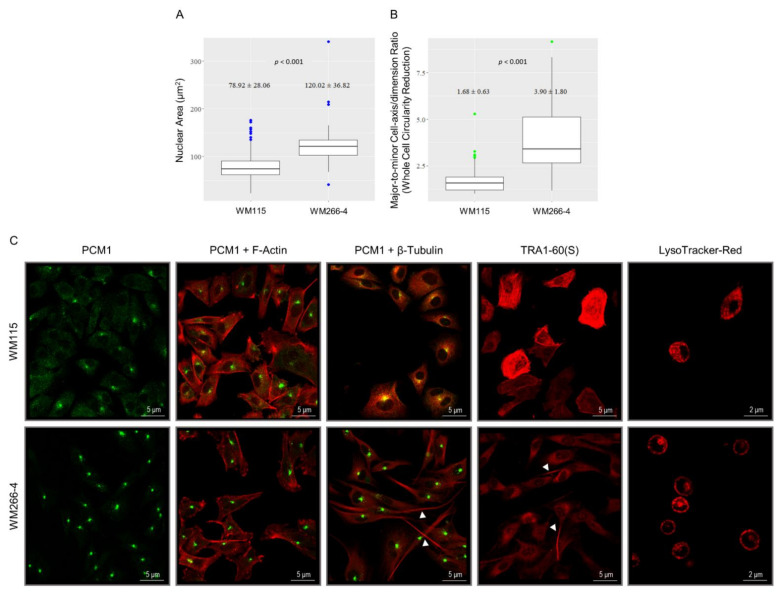
IMC program implementation in BRAF^V600D^ melanoma cells requires re-modeling of cytoskeleton architecture. (**A**,**B**) (Bio-)metrical features of WM115 (primary) and WM266-4 (metastatic) melanoma cell size and morphology/geometry using vimentin-derived patterning (Figure 2A). (**A**) “Nuclear Area” (size) (μm^2^). (**B**) “Major-to-minor cell-axis/dimension ratio” (whole cell circularity reduction). Spindle-like (elongated) “geometrical” shapes fit the fibroblast-like phenotypes of WM266-4 metastatic melanoma cells. (**C**) Single immunofluorescence profiles of “PCM1” and “TRA1-60(S) (PODXL)” proteins in WM115 (primary) and WM266-4 (metastatic) melanoma cells. Double (immuno)fluorescence patterns of “PCM1 and F-Actin”, and “PCM1 and β-Tubulin” proteins, with “β-Tubulin” (red) and “PCM1” (green) being detected via antibody-based fluorescence, and “F-Actin” being identified by rhodamine-phalloidin staining (red) in WM115 (primary) and WM266-4 (metastatic) melanoma cells. The yellow color is derived from the merger of green and red colors. Fluorescence profiles of lysosomes distribution (red) in WM115 (primary) and WM266-4 (metastatic) melanoma cells. Arrowheads: “β-Tubulin” (MT)-positive, lengthy, Invadopodia, and “TRA1-60(S)”-positive Invadopodia. “F”: Filamentous. “β”: beta. Scale bars: 2 or 5 μm.

Deregulations of cell mechanics, cell movement, cell geometry, cell shape, and cell morphology dynamics and changes are associated with various pathological states including cancer initiation, progression, and metastasis [143,144,145,146,147,148]. Therefore, WM115 (primary) was compared to WM266-4 (metastatic) melanoma cells regarding their whole cell “geometrical” shape (e.g., circular, or elongated) and nuclear area size using vimentin pattern staining (Figure 2) as a structure boundary marker (Figure 5). All measurements were made in Fiji, an open-source platform for biological image analysis [149]. Nuclear areas were measured using the corresponding measurement option, while for whole cell circularity assessment, the major-to-minor axis/dimension ratio of the ellipse fitted around the structure was carefully evaluated, with the observed differences statistically analyzed by the two-tailed *t*-test. Surprisingly, the nuclear area size of WM266-4 (120.02 ± 36.82 μm^2^) presented ~1.5× (×: fold) larger than the WM115 (78.92 ± 28.06 μm^2^) respective one with an extreme value of more than 300 μm^2^ being exclusively observed in WM266-4 metastatic melanoma cells (Figure 5A). Importantly, whole cell “geometrical” shape and morphology proved significantly more elongated (~2.3×) for WM266-4 (3.90 ± 1.80) compared to WM115 (1.68 ± 0.63) cells, with WM266-4 exhibiting the comparatively higher variation and the most extreme cell elongation ratio/value (Figure 5B). It may be that the nucleus size and cell geometry differences (e.g., circular versus elongated shapes) justify the different number of (single) proteins identified in WM115 (*n* = 3955) (Table S1) and WM266-4 (*n* = 6681) [35] proteomic maps (see also Figure 10A), suggesting the addiction of BRAF^V600D^ metastatic melanoma disease to increased nucleus size (e.g., ~1.5×), elongated cell shape (reduced circularity) (e.g., ~2.3×), and elevated proteome content (e.g., ~1.7×).

Besides vimentin (Figure 2), actin-, and tubulin-based cytoskeleton networks are also critically implicated in the control of cell shape, movement, and invasion [150,151,152,153,154]. Both WM115 (44.00; 31.00) (Table S1) and WM266-4 (22.14; 76.06) [35] proteomic catalogues contain the *ACTR2*/ARP2 and *ACTR3*/ARP3 proteins (see also Table S10), which serve as major nucleators of branched-actin networks (e.g., in Lamellipodia) [155,156,157]. However, WM115 (primary) melanoma cells seem to exclusively express ARPIN (2.50) (Table S2), a negative regulator of ARP2/3 activity [155,158], while WM266-4 (metastatic) melanoma cells can specifically synthesize *WAS*/WASP (0.00) and ABI2 (15.11) (Table S3), two essential components of the ARP2/3 activation machinery [155,157,159]. Thereby, upregulating ARPIN or/and targeting the ARP2/3-mediated F-actin network-branching process may compel BRAF^V600D^ metastatic melanoma cells to lose their migratory and invasive capacities.

Since podocalyxin (PODXL) behaves as an EMT-induced protein, mediates cancer cell extravasation, and directly interacts with ezrin [160], an F-actin-binding protein [161], we next examined its immunofluorescence patterning in WM115 and WM266-4 cells via utilization of a mouse monoclonal antibody against TRA1-60(S) that most likely recognizes PODXL [162,163]. Surprisingly, in contrast to WM266-4, a relatively small number of WM115 cells were characterized by extremely strong TRA1-60(S)/PODXL (PODXL) expression and filamentous-like organization, with several invadopodia being enriched by PODXL accumulation, specifically in WM266-4 cell (sub-)populations (Figure 5C). Interestingly, SOX2-like DNA-binding motifs could be in silico recognized in the (distal) respective promoters (or enhancers) of *ARPIN* and *PODXL* human genes (data not shown), thereby dictating the presumable ability of SOX2 to transcriptionally (over-)activate the *ARPIN* and *PODXL* genes in WM115 primary melanoma cells (see also Figure 4). It seems that PODXL represents a novel EMT marker in primary melanoma (e.g., WM115) with its downregulated levels in metastatic melanoma (e.g., WM266-4) pointing out the activation of a hybrid EMT/MET program in the advanced disease. In accordance with a previous report [160], PODXL may promote extravasation and thus subsequent transition from primary to metastatic state(s) of melanoma cells via ezrin-mediated rearrangement(s) of the actin cytoskeleton. Of note, since PODXL can be antigenically modified in retinoic acid (RA)-treated cells [162], we herein suggest the engagement of RA-dependent antigenic aberration(s) of PODXL protein in WM266-4 cells (Figure 5C). A retinoic-acid receptor RXR-gamma (RXRG) signaling axis has recently been shown to drive the emergence of a (mutant *BRAF*) melanoma cell population that confers treatment resistance, while targeting of RXR signaling holds strong promise for delaying or obviating melanoma relapse [164]. Remarkably, the nuclear receptor RXRG (5.70) was exclusively detected in the WM266-4 proteomic map (Table S3, [35]), likely indicating the RXRG-mediated antigenic compromise and thereby functional loss of PODXL protein in metastatic (e.g., WM266-4) melanoma cells. Pharmacological inhibition of RXR activities by the selective and potent RXR antagonist HX531 [164,165], in combination with clinically approved chemotherapy schemes, may prove beneficial for the successful management of metastatic and chemoresistant BRAF^V600D^ melanoma.

Importantly, β-tubulin-based microtubules (MTs) are subjected to a structural re-organization during the primary (e.g., WM115) to metastatic (e.g., WM266-4) melanoma cell transition process (Figure 5C). In contrast to WM115, WM266-4 cells exhibit fibroblast-like “geometrical” shapes and formation of invadopodia that are structurally supported by the metastatic MT network. Microtubule organizing centers (MTOCs) can be recognized in both cell types, albeit with rather distinct architectures (Figure 5C). Remarkably, since *TUBB4A*/TBB4A (tubulin beta-4A chain) was identified as the most abundant protein (348.32) exclusively expressed in WM266-4 cells (Table S3, [35]), it could presumably lead to a metastasis-specific re-modeling of the MT network in melanoma disease. Interestingly, knockdown of *TUBB4B* gene (TBB4B: see Table S10) can sensitize lung cancer cells to vincristine-induced apoptosis [166,167], thereby supporting a role of TBB4(A)-mediated chemoresistance in BRAF^V600D^ metastatic melanoma (e.g., WM266-4) cells.

Centrosome (comprises two centrioles and pericentriolar material) serves as a dominant MTOC, which directs the assembly of the MT cytoskeleton that is crucial for cell division [168,169,170]. To investigate its contribution to BRAF^V600D^ melanoma metastasis, WM115 and WM266-4 proteomic collections were analyzed regarding their centrosomal protein contents. WM115 (primary melanoma) cells were shown to contain the *CEP170*/CE170 (58.86), *CEP112*/CE112 (1.65), PCNT (1.61), CEP85 (0.00), *CEP120*/CE120 (0.00), *CEP170B*/C170B (0.00), and CETN2 (0.00) centrosomal (and centrosome-related) components (Table S1). WM266-4 (metastatic melanoma) cells were presented to carry the *CEP170*/CE170 (28.14), PCM1 (22.65), *CEP350*/CE350 (7.76), CETN2 (4.50), CCSAP (4.43), *CEP170B*/C170B (3.84), *CEP162*/CE162 (3.13), *CEP131*/CP131 (2.38), NIN (2.34), CEP89 (1.88), *CCP110*/CP110 (0.00), CEP70 (0.00), CEP85 (0.00), *CEP85L*/CE85L (0.00), *CEP152*/CE152 (0.00), *CEP192*/CE192 (0.00), *CEP290*/CE290 (0.00), *CEP295*/CE295 (0.00), CNTRL (0.00), PCNT (0.00), POC1B (0.00), and *SPICE1*/SPICE (0.00) centrosomal (and centrosome-associated) proteins [35]. These data strongly suggest the metastasis-dependent re-composition of centrosome structure in mutant BRAF melanoma settings.

Given the critical role of PCM1, a major component of centriolar satellite(s) (CS), in the assembly of centrosomal proteins and MT network organization [171,172], we next examined its immunofluorescence patterning in WM115 and WM266-4 cells (Figure 5C). Both cell types proved positive for strong PCM1 immunostaining, with WM115 featuring a notably more scattered cytoplasmic profiling compared to the WM266-4 respective one (Figure 5C). Large-sized “specks” neighboring cell nuclei (in WM115 and WM266-4) may derive from a PCM1 polymerization process, whereas small-sized “specks” dispersed in the cytoplasm (in WM115) can presumably result from a PCM1 oligomerization, or even dimerization, mechanism. The lack of small-sized (and low signal) scattered “specks” in WM266-4 cytoplasm (Figure 5C) seems to serve as a novel metastatic biomarker for BRAF^V600D^ human melanoma. Likewise, the absence of PCM1 and β-tubulin (MT) co-localization patterns (yellow coloring) in WM266-4 cells (Figure 5C) could also be used as a valid indicator for metastatic melanoma development. It may be the different PCM1 post-translational modifications (e.g., phosphorylation) or diverse transcript splice variants that control the distinct PCM1 topology and interactivity, and CS localization between primary (WM115) and metastatic (WM266-4) melanoma cells. Accordingly, it previously described the formation of “PCM1 granules” being (besides concentrated around centrioles) scattered throughout cell cytoplasm and directed by PCM1 self-aggregation that is regulated in a cell cycle-dependent manner [172].

Since lysosomal motilities are mechanistically associated with MT dynamics and homeostasis [173,174,175,176], we subsequently studied the intracellular topology profiles of lysosomes in WM115 (primary) and WM266-4 (metastatic) melanoma cells (Figure 5C). LysoTracker-Red-stained lysosomes seem to undergo a significant re-organization of their topology during the transition process from primary (e.g., WM115) to metastatic (e.g., WM266-4) melanoma state(s). Notably, the number of cells featuring a “cortical”, peripheral (plasma membrane underlying), localization, and distribution of (acidified) lysosomes is markedly increased in WM266-4 (metastatic) compared to WM115 (primary) melanoma cells (Figure 5C). This indicates the mechanistic coupling of MT-dependent lysosome trafficking/positioning to hybrid EMT/MET-driven metastasis during BRAF^V600D^-positive melanomagenesis. Although peripheral scattering, as opposed to perinuclear clustering, of lysosomes has been associated with alterations in mTORC1-kinase activities, the mechanism that links lysosome positioning to mTOR signaling still remains elusive [173]. Altogether, the anterograde (toward cell periphery) movement of lysosomes may promote the IMC program in BRAF^V600D^ melanoma environments.

### 3.6. Molecular Modeling of a KIAA0930-VIM Gene-Fusion Product Exclusively Identified in WM266-4 Cells

Since vimentin has herein been proven as the most abundantly expressed (EMT) proteomic component in both WM115 (Table S1) and WM266-4 [35] cells (see also Table S10), its mutation-driven aberrant function(s) may critically contribute to the IMC process in BRAF^V600D^-dependent melanoma. Hence, by engaging a computational platform derived from the “Cancer Dependency Map” project (Cancer Cell Line Encyclopedia) of Broad Institute (MIT-Harvard University; Cambridge, MA, USA) [62,63,64,65], we systemically compared the mutational signatures of WM115 (primary) with WM266-4 (metastatic) melanoma cell ones (access day: 14 September 2020). WM115 cells were presented to contain 535 gene mutations (e.g., single/double nucleotide polymorphisms, insertions, and deletions) (Table S4), whereas WM266-4 cells were shown to carry 531 mutations (of similar type) (Table S5) with 114 and 110 of them being exclusively detected in WM115 and WM266-4 melanoma cells, respectively (Tables S6 and S7) (see also Figure 10). Furthermore, 15 fused genes were recognized in WM115 (Table S8), while 132 fused genes could be described in the WM266-4 (Table S9) cells (see also Figure 10), thus indicating the major importance of multiple gene-fusion product functionalities to the transition process from primary to metastatic melanoma state(s).

Surprisingly, WM266-4 (metastatic) but not WM115 (primary) melanoma cells proved to bear two *VIM*-related gene fusions: (a) a *KIAA0930*-*VIM* and (b) a *VIM*-*OGFOD3*, with *KIAA0930-VIM* being the only one to presumably result in functional protein product(s) (data not shown). KIAA0930 (K0930/C22orf9) represents a hitherto, uncharacterized protein (UniProtKB—Q6ICG6—K0930_Human) whose cognate coding gene has been recently suggested to serve as a novel candidate for lung cancer risk [177]. Employment of the bioinformatics tool “MOTIF: Searching Protein Sequence Motifs—Genome Net” (MOTIF Search) revealed that the human KIAA0930 protein isoform of 409 amino acid residues (“aa”) derived from the *KIAA0930-201* (Ensembl Transcript ID: ENST00000251993.11) transcript splice variant possesses a DUF2045 (PF09741.10) domain that embraces the “E-x-x-C-V-x-L-x-x-x-D-x-x-x-[S/T]-x-x-[G/I]-[V/I]-x-[F/Y]-x-x-[S/T]” (x: any “aa”) novel motif, which can only be detected (via MOTIF Search) in the KIAA1712/CEP44 centrosomal protein (Figure S2). CEP44 plays important roles in centrosome cohesion and linker (holds the duplicated centrosomes together) assembly [178], while it ensures the formation of centriole wall required for centriole-to-centrosome conversion [179].

Interestingly, besides the DUF2045 domain, KIAA0930 (409 “aa”) contains areas characterized by regular spacing of mainly hydrophobic (non-polar) “aa” such as (a) “A_-6-_L_-6-_L_-6-_S_-6-_A_-6-_V”, (b) “L_-6-_L_-6-_A_-6-_G”, and (c) “G_-6-_T_-6-_L_-6-_L_-6-_L_-6-_F_-6-_T”. Likewise, vimentin (*VIM-201*; Ensembl Transcript ID: ENST00000224237.9; 466 “aa”) is also presented with leucine (“L”)-rich areas, with “L” following a repeated spacing such as (a) “L_-6-_Y_-6-_L_-6-_L_-6-_L”, (b) “A_-6-_F_-6-_A_-6-_L_-6-_L_-6-_L”, and (c) “A_-6-_Y_-6-_L_-6-_L_-6-_L_-6-_M”. This “L”-specific regular spacing highly resembles the leucine-zipper (LZ) motif, which facilitates transcription factor dimerization that enables the dimer’s binding onto promoter element(s) of target genes [180,181,182,183]. Thereby, the LZ motif(s) of vimentin may directly interact (e.g., via physical heterodimerization) with the one(s) of KIAA0930, causing the putative KIAA0930-vimentin fusion/hybrid protein to obtain a novel conformation, function, and topology, and to also recruit new partners. Alternatively, the LZ-like motif(s) could not make physical contacts (e.g., lack of heterodimerization) in between vimentin and KIAA0930, but in the context of KIAA0930-vimentin might provide an energetically favorable hybrid protein conformation that likely triggers IMC in BRAF^V600D^-dependent melanoma cells.

Hence, we next attempted via an advanced computational approach to construct structural models for human vimentin, KIAA0930, and KIAA0930-vimentin hybrid protein. Although experimental data has been reported only for vimentin, these concern small fragments of the protein and not its complete “aa” sequence. Molecular models were herein built by the I-TASSER server, providing as input the sequence of each examined protein. Quality assessment of each protein model (by I-TASSER) was performed by calculation of the C-score, the template modeling score (TM-score), and the root mean square difference (RMSD). C-score is a confidence score, whose value typically ranges from −5.00 to +2.00. A high value of C-score indicates high confidence in the model. The TM-score represents a scale for measuring the structural similarity in between two proteins with different tertiary structures. A value of TM-score over +0.50 indicates the correct topology of a predicted model, while a value below +0.17 points out random similarity.

**Figure 6:**
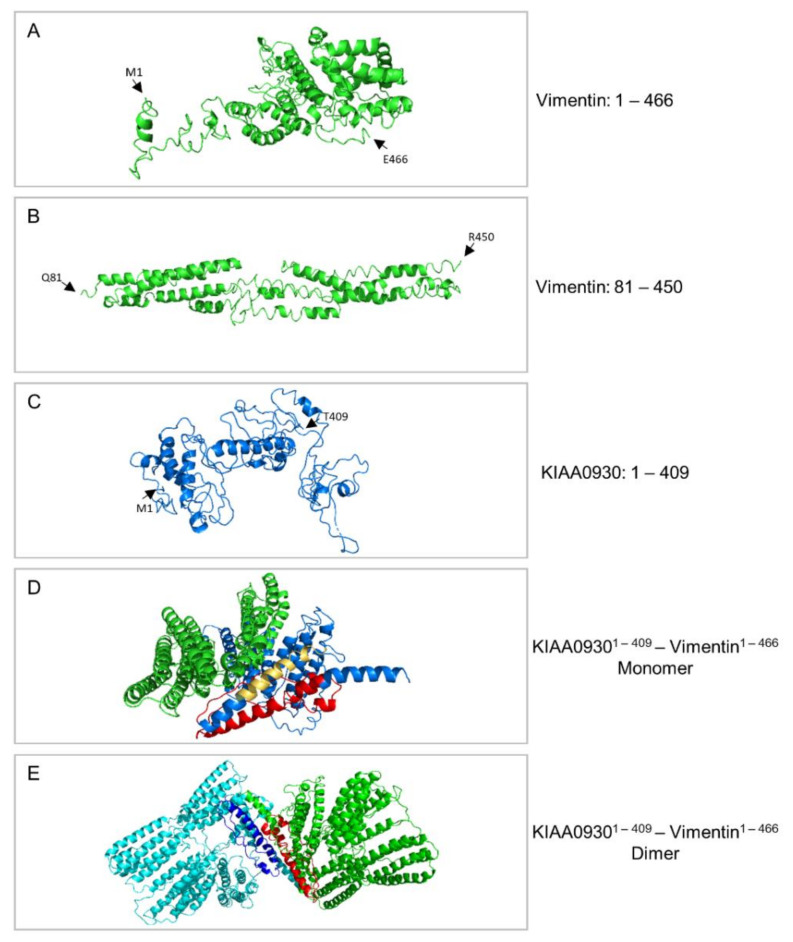
Structural models of vimentin, KIAA0930, and KIAA0930-vimentin hybrid proteins. (**A**) Structural model of the human vimentin (*VIM-201*; ENST00000224237.9; amino acid residues: 1–466). (**B**) Model of a human vimentin in silico “truncated” version (amino acid residues: 81–450). Residues 1–80 and 451–466 that correspond to the amino- (“N”) and carboxyl- (“C”) terminus of the full-length vimentin, respectively, were not used, since they proved to obtain disordered conformations (**A**). (**C**) Structural model of the human KIAA0930 protein (*KIAA0930-201*; ENST00000251993.11; amino acid residues: 1–409). (**D**) Structural model of the KIAA0930-vimentin hybrid, monomer, protein (amino acid residues: 1–409^(KIAA0930)^/1–466^(Vimentin)^). The domain that corresponds to the KIAA0930 protein is colored in blue. Residues 22–99 (colored in red) and 111–132 (colored in orange/yellow) contain motifs that share characteristics with leucine zippers (LZs). (**E**) Theoretical model of a KIAA0930-vimentin hybrid protein homodimer derived from the docking experiments. In each monomer, residues 22–99 are colored in red and blue, respectively, and are located at the interface of interacting monomers. “M”: Methionine. “E”: Glutamic Acid. “Q”: Glutamine. “R”: Arginine. “T”: Threonine.

In the case of vimentin, two different molecular models were herein built. The first one corresponded to its full-protein sequence (1–466 “aa”), with the C-score, TM-score, and RMSD obtained values being measured as −2.90, 0.38 ± 0.13, and 14.3 ± 3.8 Å, respectively (Figure 6A), clearly indicating that the predicted model was not structurally accurate and valid. Notably, model inspection (and I-TASSER results) foresaw conformational disorders at both amino- (“N”) and carboxyl- (“C”) termini of full-length vimentin (Figure 6A). However, an in silico truncated form of vimentin (81–450 “aa”) that was missing its presumably disordered parts produced a model with −1.34, 0.55 ± 0.15, and 9.7 ± 4.6 Å C-score, TM-score, and RMSD measured values, respectively, thus indicating the model’s structural reliability and accuracy (Figure 6B) compared to the full-length protein-derived one (Figure 6A). Interestingly, the ^81^vimentin^450^ modeled protein seems to adopt a fibrillar structure, which nicely justifies its ability to self-polymerize and generate long-length fibers (filaments) in vivo. Similar to the full-length vimentin (Figure 6A), the KIAA0930 (1–409 “aa”) molecularly modeled protein was also characterized by unsatisfied measurements with the C-score, TM-score, and RMSD being calculated at −2.93, 0.38 ± 0.13, and 14.0 ± 3.9 Å, respectively (Figure 6C), strongly suggesting uncertainty for the model’s fidelity and trust. Surprisingly, the KIAA0930-vimentin hybrid protein, which in silico embraces both the full-length KIAA0930 (1–409 “aa”) and vimentin (1–466 “aa”) protein sequences, led to a notably satisfactory molecular model, with the C-score, TM-score, and RMSD parameters obtaining −0.22, 0.68 ± 0.12, and 9.1 ± 4.6 Å measured values, respectively (Figure 6D), thereby dictating the model’s accuracy and reliability. Next, via employment of the automated protein docking server ClusPro, docking experiments were suitably conducted to examine the homodimerization capacity of the KIAA0930-vimentin hybrid monomer protein. Indeed, as illustrated in Figure 6E, KIAA0930-vimentin proved capable of self-dimerizing through a molecular interface that contained the KIAA0930-specific LZ-like motif “A_-6-_L_-6-_L_-6-_S_-6-_A_-6-_V”.

An unexpected and surprising finding of the present study is the ability of KIAA0930 and vimentin proteins to obtain structurally robust conformations exclusively at their hybrid state context, thus indicating that KIAA0930-vimentin likely acquires novel properties concerning intracellular functionality, interactivity, regulation, and topology. Since *KIAA0930*/K0930 (KIAA0930) (3.88) could be solely identified in the WM266-4 proteomic map (Table S3, [35]), the *KIAA0930*-*VIM* gene-fusion product(s) may critically contribute to the IMC process of BRAF^V600D^-dependent human melanomagenesis. Given that the DUF2045-accomodated “E-x-x-C-V-x-L-x-x-x-D-x-x-x-[S/T]-x-x-[G/I]-[V/I]-x-[F/Y]-x-x-[S/T]” novel motif, besides KIAA0930, can also be recognized in the CEP44 centrosomal protein (Figure S2), it can presumably serve as a dimerization signal for KIAA0930 and CEP44 specific interactions. If so, KIAA0930-vimentin (only in its structured conformation) may direct CEP44 away from its target, the centrosome, likely compromising the fidelity of mitotic division and promoting incidents of chromosomal heterogeneity. Intriguingly, LRRC45, a component of centrosomal linker [184], carries a new motif of regularly spaced “L”, the “L_-12-_L_-12-_L_-12-_L_-12-_L” sequence (data not shown), which through its putative interaction(s) with the LZ-like motifs of structured KIAA0930-vimentin hybrid protein may be depleting centrosome(s) from LRRC45, further deregulating cell-division and chromosomal-segregation processes. Alternatively, KIAA0930-vimentin could recruit centrosomal components (or even ventrosomes) onto vimentin cytoskeleton filaments, probably facilitating invadopodia formation in metastatic melanoma (e.g., WM266-4) cells via coordinated and synergistic activities of vimentin- and MT-based networks. Accordingly, it was previously reported that the PCM (pericentriolar material) of a centrosome appears to possess attachment sites for vimentin intermediate filaments (VIFs) and perinuclear VIFs can infiltrate the PCM [185]. Our inability to detect the putative KIAA0930-vimentin hybrid protein in a western blotting-derived vimentin profiling (Figure 2) suggests the major antigenic alteration(s) of the hybrid protein or/and the extremely low rate(s) of gene-fusion and hybrid protein synthesis events. The systemic examination of tumor biopsies derived from patients having been affected by primary or metastatic BRAF^V600D^-positive melanoma(s) (before and after drug treatment) for *KIAA0930*-*VIM* gene-fusion incidents (Table S9) may prove beneficial for the successful management of advanced disease in the clinic.

### 3.7. Signal Transduction SignatuRes. in BRAF^V600D^-Dependent Melanoma Undergoing Metastasis

**Figure 7:**
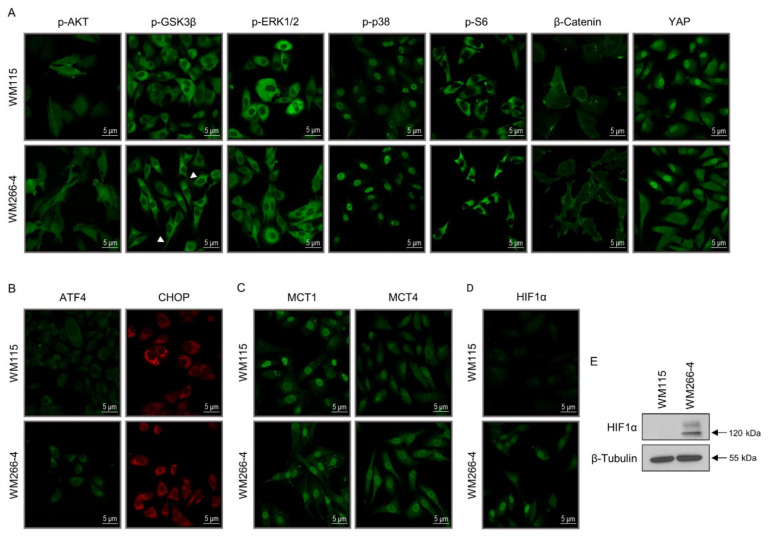
Signaling signatures of the IMC program in BRAF^V600D^-dependent melanomagenesis. (**A**) Immunofluorescence patterns of the “p-AKT”, “p-GSK3β”, “p-ERK1/2”, “p-p38 (MAPK)”, “p-S6”, “β-catenin”, and “YAP” signal transduction-related proteins in WM115 (primary) and WM266-4 (metastatic) melanoma cells. “p”: Phosphorylation. “β”: Beta. Arrowheads: “p-GSK3β”-positive invadopodia. Scale bars: 5 μm. (**B**) Immunofluorescence profiles of the “ATF4” and “CHOP” ER-stress/UPR-related proteins in WM115 (primary) and WM266-4 (metastatic) melanoma cells. “ER”: Endoplasmic reticulum. Scale bars: 5 μm. (**C**) Immunofluorescence patterns of the “MCT1” and “MCT4” Monocarboxylate (Lactate) transporters in WM115 (primary) and WM266-4 (metastatic) melanoma cells. Scale bars: 5 μm. (**D**) Immunofluorescence profiles of the “HIF1α” transcription factor in WM115 (primary) and WM266-4 (metastatic) melanoma cells. “α”: Alpha. Scale bars: 5 μm. (**E**) Western blotting examining the “HIF1α” expression profiles in WM115 (primary) and WM266-4 (metastatic) human BRAF^V600D^-positive melanoma cells. “β-Tubulin” served as the protein of reference (control). “α”: Alpha. “β”: Beta. Molecular weights of the “HIF1α” and “β-Tubulin” (major) proteins are shown by numbers at the right side of each respective panel. (**E**) Protein quantification values (in bar-chart format) are shown in Figure S1.

Oncogenic signaling is considered as a major mechanistic hallmark of cancer, while kinase deregulation has been firmly demonstrated to essentially contribute to tumor initiation, progression, and metastasis [186,187]. Thereby, we herein analyzed the phosphorylation state and localization pattern of critical kinases, whose activation or inactivation processes could be tightly associated with BRAF^V600D^ melanoma metastasis. Surprisingly, it proved that the obtained immunofluorescence profiles of p-AKT [188,189,190] (cytoplasmic/membranous), p-GSK3β (an AKT target) [188,190,191,192] (cytoplasmic: punctuated pattern and adjacent to nucleus topology; Invadopodia), p-ERK1/2 [193,194,195,196] (cytoplasmic), p-p38 MAPK (p-p38) [193,197,198] (nuclear), and p-JNK [193,197,198] (absent) (data not shown) phosphorylated kinases did not significantly differ in between WM115 (primary) and WM266-4 (metastatic) melanoma cells (Figure 7A). Likewise, p-S6, an important mTOR kinase signaling effector [188,190,193,199,200,201], was presented with similar patterning (cytoplasmic) in primary (e.g., WM115) and metastatic (e.g., WM266-4) melanoma cells (Figure 7A). In the same direction, *CTNNB1*/CTNB1 (β-catenin) (28.15; 37.60) (Table S1, [35]) (see also Table S10), a downstream mediator of WNT-driven signaling [202,203,204], was rather equally expressed and localized (membranous), albeit at low to moderate levels, in between WM115 and WM266-4 melanoma cells (Figure 7A). YAP1 (YAP) (35.87; 11.49) (Table S1, [35]) (see also Table S10) transcriptional co-activator, a pivotal effector of HIPPO signaling (off) pathway [205,206,207], was shown to be compartmentalized and highly accumulated in melanoma nuclei of both cell types. However, YAP-positive nuclei could be recognized in the majority of WM115 cells, whereas they were numerically reduced in WM266-4 cells (Figure 7A), thus denoting the HIPPO signaling heterogeneity mainly at the metastatic state. Taken together, it seems that both primary and metastatic melanoma cells have activated the AKT/GSK3-, ERK-, p38-, and mTOR-specific signaling pathways, likely due to insulin, growth factor, or/and cytokine signaling networks that can operate in an autocrine type, and, thus autonomous and self-sustained manner. Of note, the WM266-4 proteomic map was exclusively featured with detectable contents of FGF2 (0.00), FGF13 (0.00), IL12A (0.00), IL17C (0.00), and IL17F (0.00) signaling receptor ligands (Table S3, [35]), strongly supporting the self-sustained survival, proliferation, and growth capacity of metastatic melanoma cells. Furthermore, both melanoma cell types were shown to carry mutant forms of BRAF (V600D; V600E), HGF (R197C), PDGFA (L164fs), and RPTOR (R788C) signaling components (Tables S4 and S5) (see also Table S13), further underpinning the autonomous and deregulated functionalities of oncogenicity-controlling signaling networks in primary and metastatic melanomas.

Since p38 kinase can be activated by diverse micro-environmental signals including oxidative stress and DNA damage [193,197], WM115 and WM266-4 cells may have already been exposed to cues promoting ROS (reactive oxygen species) production, hypoxic growth, and genotoxic responses, therefore obligating the activated (e.g., phosphorylated) p38 kinase to massively accumulate in the BRAF^V600D^ melanoma nucleus (Figure 7A). Although the expression of activated AKT1 (AKT) can promote the development of melanoma metastases in certain oncogenic settings with mTOR signaling (downstream of AKT) also being implicated in driving metastasis [208], the lack of activated AKT- and mTOR-patterning differences in between WM115 (primary) and WM266-4 (metastatic) melanoma cells (Figure 7A) likely indicates the genetically contextual nature of AKT- and mTOR-mediated signaling during the IMC course of BRAF^V600D^-dependent human melanomagenesis. It may be that the specific mutational signatures of WM115 (e.g., PIK3R1^R642*^: AKT regulator) (Table S6) and WM266-4 (e.g., MMP9^G100E^: IMC modulator) (Table S7) cells render an activated kinase form (e.g., p-AKT) to act as an oncogenic driver for primary melanoma or/and metastatic melanoma development in a BRAF^V600D^ genetic background. According to their previously reported roles in the acquisition of chemoresistance [190,193,198], activated AKT, ERK, p38, and mTOR kinase transduction courses can likely provide WM115 and WM266-4 cells with certain levels of tolerance to specific regimens targeting BRAF^V600D^-dependent melanomagenesis.

Likewise, given the importance of YAP transcriptional actions to cancer chemoresistance [206,209,210], its nuclear compartmentalization in both primary (e.g., WM115) and metastatic (e.g., WM266-4) melanoma cells strongly suggests the HIPPO^Off^/YAP^On^ signaling axis as a major contribution to genetically directed chemoresistance in the advanced disease. Since the protein products of *AMOTL1* and *AMOTL2*, two typical YAP-target genes [205,206], could both be identified in the WM115 (2.74; 0.00) (Table S1) and WM266-4 (0.00; 0.00) [35] proteomic collections (see also Table S10), a YAP-specific (HIPPO^Off^) molecular signature seems to be tightly associated with the development of BRAF^V600D^-positive melanomagenesis. The significant heterogeneity of YAP-positive nuclear localization pattering (e.g., YAP^+^ and YAP^−^ nuclei) herein observed solely in WM266-4 cells (Figure 7A) mechanistically reflects the HIPPO signaling heterogeneity (e.g., HIPPO^Off^ and HIPPO^On^, respectively) and dictates the critical role(s) of HIPPO^Off^/YAP^On^ signaling pathway in the IMC program during BRAF^V600D^-dependent melanoma formation. Given the principal implication of YAP activity in the transcriptional control of EMT [205,206,211], and the WM266-4-specific nuclear YAP profiling heterogeneity (Figure 7A), it seems that a YAP-dependent hybrid EMT/MET program is required for IMC in mutant BRAF (e.g., V600D) human cutaneous melanoma(s).

Sustained activation of endoplasmic reticulum (ER)-stress sensors can endow tumor cells with greater metastatic capacity [212,213,214,215]. The unfolded protein response (UPR) adaptive mechanism that is induced to restore ER homeostasis essentially contributes to multiple steps along IMC including EMT [212,214,216]. Interestingly, it seems that the PERK-eIF2α-ATF4 signaling branch of UPR can be selectively and constitutively activated by cancer cells having undergone EMT [212,216], with PERK kinase inhibition or *ATF4* gene silencing dramatically reducing tumor (lung) metastasis ability in vivo [212,216,217]. Hence, we herein examined the immunofluorescence patterning of the ATF4 transcription factor, and its downstream *CHOP* gene-target product [212,214] in WM115 and WM266-4 cells (Figure 7B). Both ER-stress/UPR-related transcription factors ATF4 and CHOP were shown to be excluded from the nucleus, and specifically compartmentalized in the cytoplasm (Figure 7B), thus indicating the PERK-ATF4-CHOP signaling-independent transition process from a primary (e.g., WM115) to metastatic (e.g., WM266-4) melanoma state(s).

Metabolic re-programming is tightly associated with the tumor’s metastatic potential. Since the MCT1 lactate transporter (importer) plays diverse critical roles in the metastasis process [218,219,220,221,222,223], we next analyzed the cellular localization and distribution pattern of the MCT1 (*SLC16A1*/MOT1) transporter in WM115 and WM266-4 (2.70) (Table S3, [35]) melanoma cells (Figure 7C). Surprisingly, through immunofluorescence imaging, MCT1 was presented to strongly accumulate in the melanoma nucleus and not the cell membrane (as expected) for both cell types (Figure 7C). Likewise, MCT4 (*SLC16A3*/MOT4), another critical lactate transporter (exporter) of the family [220,224], also exhibited a nuclear compartmentalization pattern and an unexpected absence from the cell membrane area in both WM115 (primary) (2.55) (Tables S1 and S2) and WM266-4 (metastatic) melanoma cells (Figure 7C). Intriguingly, nuclear localization of MCT1 has also been observed in prostate cancer cells [225], soft-tissue sarcomas [226], and endometrial cancer [227]. Similarly, MCT4 was previously described to reside in the cell nucleus throughout mouse embryo pre-implantation development [228], while a weak signal profile could be detected in the nucleus of pancreatic cancer cells [229]. Given that MCT1 can promote tumor metastasis independently of its lactate-transporter activity [220,221], novel molecular functions of the MCT1 and MCT4 family members likely remain to be discovered. Thereby, the nuclear topology of MCT1 and MCT4 in primary (e.g., WM115) and metastatic (e.g., WM266-4) melanoma cells may indicate new and transporter-independent properties of these proteins, causing them to act as orchestrators of nuclear architecture or/and regulators of gene transcription during BRAF^V600D^-dependent melanomagenesis. Most importantly, nuclear MCT1 (nMCT1) and MCT4 (nMCT4) patterning could serve as a clinically reliable and valid (diagnostic) biomarker for the disease.

Aside from metabolic adaptation, hypoxic adaptation that involves hypoxia-inducible (transcription) factors (HIFs) is also a survival mechanism for tumor cells [220,230,231]. Hypoxia has proved able to activate the *SLC16A3* gene transcription in a HIF1-mediated fashion [220,232], thereby re-modeling the metabolite profiling of hypoxic cells. Depletion of oxygen (O_2_) likely leads to oxidative stress, which can cause detrimental protein misfolding that induces ER-stress and adaptive UPR [231,233]. Most importantly, hypoxia has been mechanistically linked to EMT, with HIF1 playing a major role in this oncogenic association [234,235,236]. Therefore, we herein examined the expression patterning of HIF1α transcription factor in WM115 and WM266-4 (0.00) (Table S3, [35]) melanoma cells via employment of both immunofluorescence (Figure 7D) and western blotting (Figure 7E) experimental platforms. Remarkably, in accordance with WM115 and WM266-4 proteomic maps (Tables S1–S3 [35]), HIF1α was exclusively detected in WM266-4 (metastatic), but not WM115 (primary) melanoma cells (Figure 7D,E), strongly suggesting its principal contribution to the IMC program during BRAF^V600D^-dependent melanomagenesis. Since many WM266-4 cells have presented with a strong nuclear compartmentalization pattern of HIF1α (Figure 7D), it seems that metastatic melanoma cell sub-populations can activate a HIF1α-mediated transcriptional program independently of their normoxic growth setting(s). Despite their normoxic culturing in vitro, WM266-4 melanoma cells can retain their tumor-derived hypoxic adaptations, engaging HIF1α to orchestrate this “hypoxic memory” process. Alternatively, a hypoxia-independent mechanism such as the formation of genetic alterations [237] might be activated for the localization of HIF1α in the metastatic melanoma nucleus.

In normoxic (e.g., well oxygenated) tissues, HIF1α is unstable due to a ubiquitin-dependent degradation that is mediated through hydroxylation of two critical prolines. Hydroxylated HIF1α is recognized by VHL and elongins (B and C), causing its subsequent proteasomal degradation. In hypoxic (low O_2_ tension) micro-environments, HIF1α cannot be hydroxylated and thus becomes structurally (physically) stabilized. Propyl hydroxylases (PHDs or EGLNs) use O_2_ as a low-affinity substrate to carry out the HIF1α-specific hydroxylation process, thus serving as pivotal regulators for the “switch” of HIF1α transcription factor from an “on” (non-hydroxylated) to an “off” (hydroxylated) molecular state and vice versa [231,237,238,239,240,241]. Interestingly, the WM115 proteome has been shown to carry both EGLN1 (10.48) and EGLN3 (0.00) proteins (Tables S1 and S2), while the WM266-4 proteome was presented to contain only the EGLN1 (18.66) family member [35], indicating the metastasis-induced alleviation of propyl-hydroxylation load in melanoma cells and the HIF1α subsequent stabilization, nuclear localization, and transcriptional program activation. Indeed, the identification of *IGFBP3*/IBP3 (16.79), *KRT19*/K1C19 (5.15), *PFKFB3*/F263 (3.23), *PLAUR*/UPAR (3.00), TIMP1 (1.83), *ABCB1*/MDR1 (0.00), *PFKFB4*/F264 (0.00), and SOCS3 (0.00) proteins, whose cognate genes constitute HIF1(α) targets [237,239,241,242] solely in the WM266-4 proteomic catalogue (Table S3, [35]), points out the activation of the HIF1(α) target gene repertoire in BRAF^V600D^-dependent metastatic melanoma. The HIF1α signaling pathway may be critically implicated in the control of the IMC process, chemotherapeutic resistance, and genetic/phenotypic heterogeneity of BRAF^V600D^-positive melanoma cells, thus rendering nuclear (and activated) HIF1α as a promising drug target for the advanced disease.

### 3.8. Apoptotic and Autophagic Sub-Routines in BRAF^V600D^ Primary and Metastatic Melanoma Cells

**Figure 8:**
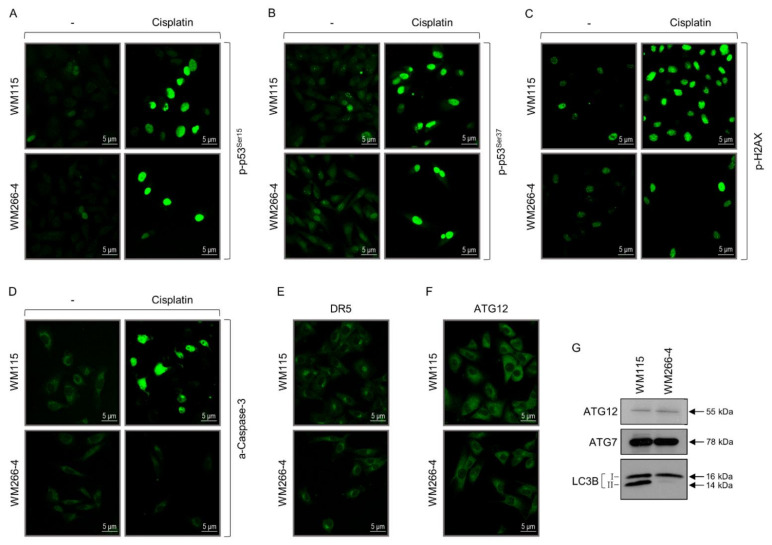
Programmed cell death sub-routines in BRAF^V600D^-positive primary and metastatic melanoma cells. (**A**) Immunofluorescence patterns of the “p-p53^Ser15^” protein in WM115 (primary) and WM266-4 (metastatic) melanoma cells, in the absence (−) or presence of cisplatin (50 μg/mL, for 24 h) chemotherapeutic agent. “p”: Phosphorylation. “Ser”: Serine. Scale bars: 5 μm. (**B**) Immunofluorescence profiles of the “p-p53^Ser37^” protein in WM115 (primary) and WM266-4 (metastatic) melanoma cells, in the absence (−) or presence of cisplatin (50 μg/mL, for 24 h). “p”: Phosphorylation. “Ser”: Serine. Scale bars: 5 μm. (**C**) Immunofluorescence patterns of the “p-H2AX” protein in WM115 (primary) and WM266-4 (metastatic) melanoma cells, in the absence (−) or presence of cisplatin (50 μg/mL, for 24 h). “p”: Phosphorylation. Scale bars: 5 μm. (**D**) Immunofluorescence profiles of the (cleaved/activated) “a-caspase-3” protein in WM115 (primary) and WM266-4 (metastatic) melanoma cells, in the absence (-) or presence of cisplatin (50 μg/mL, for 24 h). “a”: Activated. Scale bars: 5 μm. (**E**) Immunofluorescence patterns of the “DR5” transmembrane death receptor in WM115 (primary) and WM266-4 (metastatic) melanoma cells. Scale bars: 5 μm. (**F**) Immunofluorescence profiles of the “ATG12” autophagy protein in WM115 (primary) and WM266-4 (metastatic) melanoma cells. Scale bars: 5 μm. (**G**) Western blotting-mediated examination of the “ATG12”, “ATG7”, and “LC3B (I/II)” protein expression profiles in WM115 (primary) and WM266-4 (metastatic) BRAF^V600D^-dependent human melanoma cells. Molecular weights of the herein identified autophagy proteins are denoted by numbers at the right side of each respective panel. (**G**) Protein quantification values (in bar-chart format) are shown in Figure S1.

Given the essential roles of the p53 transcription factor in stress-signaling responses including HIF1α-dependent hypoxia and DNA damage [243,244,245,246,247,248,249,250,251], cancer metastasis [252,253,254,255,256,257,258], cell-cycle arrest, and apoptosis induction [247,248,249,250,251,258,259], we next investigated the phosphorylated (activated) p53 (p-p53^Ser15^ and p-p53^Ser37^) profiles in WM115 and WM266-4 melanoma cells, in the absence or presence of cisplatin (Figure 8), a DNA-damaging agent that is widely used in clinical practice for cancer chemotherapy [260,261]. A 24 h exposure of WM115 (primary) and WM266-4 (metastatic) melanoma cells to 50 μg/mL cisplatin caused the strong induction of p53 phosphorylation at the critical serine-15 (Ser15) and −37 (Ser37) residues, as demonstrated by the nuclear accumulation of p-p53^Ser15^ (Figure 8A) and p-p53^Ser37^ (Figure 8B) protein forms in both primary (WM115) and metastatic (WM266-4) melanoma cells. Remarkably, WM266-4 cells were presented with significantly reduced numbers of p-p53^Ser15^- and p-p53^Ser37^-negative nuclei compared to WM115 ones, thus suggesting their competence to proficiently sense DNA harms, and engage activated p53-mediated mechanisms to counteract the genotoxic actions of cisplatin. Since phosphorylation of the p53 transcription factor at Ser15 and Ser37 can be conducted (likely among others e.g., ATR and p38) by the activated ATM and DNAPK kinases in response to DNA damage, impairing the ability of MDM2 negative regulator to inhibit its transcriptional activity [243,244,247,258,259,262,263], it seems that metastatic (e.g., WM266-4) melanoma cells may have upregulated the ATM and/or DNAPK signaling functions to maintain genomic integrity and ensure survival capacity despite their exposure to cisplatin-induced stress.

Similarly, cisplatin (via immunofluorescence) proved able to induce strong phosphorylation of the H2AX histone (p-H2AX) in both WM115 (primary) and WM266-4 (metastatic) melanoma cells (Figure 8C). Nevertheless, WM266-4 presented markedly lower numbers of p-H2AX-negative cells compared to the WM115 respective ones in response to cisplatin, indicating the critical contribution of p-H2AX-emanated signaling to the IMC of mutant BRAF human melanoma(s) during chemotherapy. Since p-H2AX serves as a sensitive marker for DNA double-strand break (DSB) formation and a key factor for DNA-damage repair [247,249,264], its genotoxicity-sensing proficiency can generally enhance chromosomal stability and reduce phenotypic heterogeneity in metastatic state(s) of BRAF^V600D^-positive melanoma(s). However, it may be that the few p-H2AX-negative WM266-4 cells can efficiently survive and propagate in the presence of cisplatin, leading to new chemoresistant and heterogenic melanoma cell sub-populations with strong invasion and metastatic activities. Hence, restoring H2AX DNA-repair function in drug-treated cell escapers may open a new therapeutic window for the successful management of advanced melanoma(s) in the clinic.

Given the multiple roles of H2AX in apoptosis [249,265,266,267,268,269], we next investigated the imaging patterns of activated (cleaved) caspase-3 (a-caspase-3), a major effector of apoptotic cell death [247,248,270,271,272,273,274,275], in cisplatin-exposed WM115 and WM266-4 melanoma cells (Figure 8D). Strikingly, a strong a-caspase-3-positive cell staining was detected only in WM115 (primary), but not WM266-4 (metastatic) melanoma cells after their exposure to cisplatin (Figure 8D), thus indicating the metastasis-dependent development of resistance to genotoxicity-induced apoptosis in BRAF^V600D^ melanoma environments. Post-treatment, although the total number of analyzed (remaining) WM266-4 cells was significantly reduced compared to the WM115 respective one, the proportion of a-caspase-3-negative versus -positive cells proved to be notably increased in the WM266-4 (metastatic) than WM115 (primary) melanoma cells, thus pointing out the impotence of cisplatin to efficiently eliminate, via (activated) caspase-3-mediated apoptotic death, the WM266-4 cell sub-populations. There may be several a-caspase-3-negative cell escapers from cisplatin’s toxic actions (Figure 8D) that enable BRAF^V600D^ metastatic melanoma(s) to develop strong chemoresistance against the applied therapeutic regimens in the clinic.

TRAIL belongs to the ligands that activate the caspase repertoire to drive tumor cells to apoptosis. However, tumor, and especially melanoma, cells can evade TRAIL-directed apoptotic death via engagement of diverse molecular mechanisms including downregulation of its cognate TRAIL-R2 (DR5) death receptor [271,276,277,278,279]. Thereby, the DR5 immunofluorescence profiling was herein imaged in WM115 and WM266-4 melanoma cells (Figure 8E). Surprisingly, DR5 was presented with a strong cytoplasmic, but not membranous (as expected), compartmentalization patterning for both cell types with the obtained staining being asymmetrically accumulated in close to nucleus areas resembling the ER/Golgi apparatus (Figure 8E), markedly underpinning previously reported observations [277,278,280]. It seems that primary (e.g., WM115) and metastatic (e.g., WM266-4) melanoma cells can re-program their DR5-specific trafficking routes to exclude DR5 from its typical cell membrane niche, thus prohibiting TRAIL-DR5 signaling axis activation and promoting resistance to TRAIL-triggered apoptosis. Interestingly, the DR4/DR5-emanated apoptotic signaling can be selectively attenuated by the EMT program. Binding of E-cadherin (a major epithelial marker) specifically to the ligated DR4/DR5 death receptors causes augmentation of the downstream apoptotic signaling (e.g., caspase-8 activation), while depletion of E-cadherin significantly reduces sensitivity to cell death induction by TRAIL [281]. Therefore, the absence of E-cadherin from WM115 and WM266-4 cells (Figure 2) may attenuate the TRAIL-DR5-dependent apoptosis, likely begetting BRAF^V600D^ melanoma resistance to TRAIL administration. Besides its apoptotic signaling impotence, cytoplasmic DR5 (cDR5) clustering may be critically implicated in the initiation and progression of BRAF^V600D^-dependent cutaneous melanoma disease. Indeed, the TRAIL-DR5 pathway, instead of triggering apoptosis, can induce the metastatic potential of mouse melanoma cells [282]. Likewise, oncogenic KRAS can convert death receptors into metastasis-promoting receptors in colorectal cancer cells [283,284,285,286]. Taken together, we herein suggest that clustered cDR5 (in the absence of TRAIL) may act as an IMC regulator in BRAF^V600D^-positive melanoma micro-environments.

In addition to apoptosis, (macro-)autophagy also serves as a pivotal cell death sub-routine [270,272,287,288,289,290]. However, autophagy can have two opposite, contextual, functions in cancer: (a) a tumor-suppression activity via elimination of oncogenic proteins, unfolded (toxic) components, and damaged organelles, and (b) a tumor-progression activity via intracellular recycling to provide metabolism substrates for functional mitochondria maintenance [288,291,292,293,294,295,296,297,298]. Notably, activated RAS requires autophagy to maintain tumorigenesis [291,299,300], while autophagy and MAPK signaling pathways can cooperate to preserve mutant RAS cancer cell survival [301]. Hence, via employment of immunofluorescence (Figure 8F) and western blotting (Figure 8G) technical protocols, we next examined the expression levels of ATG12, ATG7, and LC3B proteins, which act as critical components of the autophagic machinery [66,272,288,290,293,294,295,302] in WM115 and WM266-4 melanoma cells (Figure 8F,G). Interestingly, both cell types proved to carry similar contents of (cytoplasmic) ATG12 (Figure 8F,G) and ATG7 (Figure 8G) autophagic proteins, with ATG7 being more strongly upregulated compared to ATG12. However, LC3B was presented with a major difference of its expression profiling in between WM115 (primary) and WM266-4 (metastatic) melanoma cells. Strikingly, although the LC3B-I (16 kDa) high(er) mobility form could be equally detected in both WM115 and WM266-4 cells, the LC3B-II (14 kDa) low(er) mobility form was significantly downregulated in WM266-4 metastatic melanoma cells (Figure 8G). Since LC3B-II serves as a major indicator/regulator of mammalian autophagy [272,288,290,293,294,295,303,304,305,306], it seems that WM115 primary melanoma cells (LC3B-II^High^) have activated a functional program of basal (constitutive) autophagy, whereas WM266-4 metastatic melanoma cells (LC3B-II^Low^) have markedly attenuated their constitutive autophagy program.

Altogether, our data indicate the opposite role(s) of constitutive autophagy in primary and metastatic human melanomagenesis. In a BRAF^V600D^ genetic background, constitutive autophagy may be required for primary melanomagenesis, but it is likely dispensable or harmful for the metastatic stage(s) of the disease. Remarkably, inhibition of autophagy can drive the emergence of breast cancer stem cells from metastatic dormancy by inducing and stabilizing *PFKFB3*/F263 expression [307]. Since *PFKFB3*/F263 (3.23) was exclusively identified in the WM266-4 proteomic map (Table S3, [35]) and *PFKFB3* represents a bona fide target gene of the HIF1(α) transcription factor [237,239,241,242], which is solely expressed in WM266-4 cells (Figure 7) (Table S3, [35]), a HIF1α-F263 signaling axis can presumably control the metastatic dormancy awakening of mutant BRAF (e.g., V600D) human melanoma (NC) stem cells. Pharmacologically inducing autophagy [297] and systemically administering vemurafenib (BRAF inhibitor) may prove therapeutically beneficial for LC3B-II^Low^, HIF1α^High^, and BRAF^V600D^-positive metastatic melanoma(s). Alternatively, simultaneous targeting of F263 [308,309,310] (or/and HIF1 [237,311]) and BRAF^V600D^ [17,18,19,20,21,22,23,312] could likely result in the efficient elimination of awakening (e.g., dividing) melanoma (NC) stem cells and thus (long-term) significant improvement of advanced disease therapeutic responses in the clinic.

### 3.9. Targeted Drugging Efficacy against WM115 and WM266-4 Melanoma Cells Depends on Their IMC States

**Figure 9:**
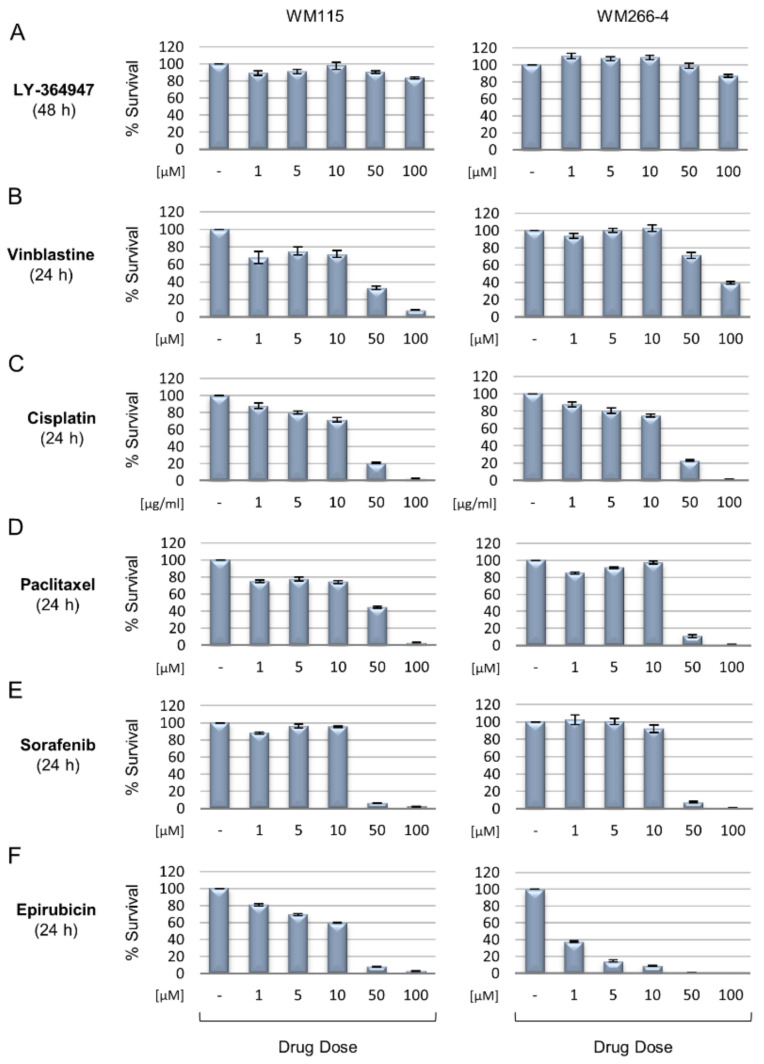
IMC states dictate targeted drugging potency in BRAF^V600D^-dependent melanoma cells. (**A**–**F**) MTT assays in bar-chart format demonstrating survival percentages (%) of WM115 (primary) and WM266-4 (metastatic) human BRAF^V600D^-positive melanoma cells, in response to: (**A**) “LY-364947” (0–100 μM, for 48 h), (**B**) “Vinblastine” (0–100 μM, for 24 h), (**C**) “Cisplatin” (0–100 μg/mL, for 24 h), (**D**) “Paclitaxel” (0–100 μM, for 24 h), (**E**) “Sorafenib” (0–100 μM, for 24 h), and (**F**) “Epirubicin” (0–100 μM, for 24 h).

Given the ability of LY-364947, a specific TGF-β signaling inhibitor [126,130,313,314,315,316], to remarkably inhibit the migratory (and invasion) capacity of both WM115 (primary) and WM266-4 (metastatic) melanoma cells in vitro (Figure 3), we next investigated its cytotoxic potency against the two cell types via employment of a MTT survival assay-based technology (Figure 9). Notably, 48 h treatment with LY-364947 proved unable to drive WM115 and WM266-4 cells to death (Figure 9A), indicating the distinct TGF-β signaling roles in controlling melanoma cell survival (Figure 9) versus motility/migration activity (Figure 3) in a time-course of 48 h. Nevertheless, many cell death incidents were observed exclusively in WM115 cells upon their exposure to LY-364947 (100 μM) for 72 h (Figure 3), thereby revealing the inhibitor’s power to impair both migratory/invasion and survival/growth abilities of BRAF^V600D^ primary melanoma cells in response to its (LY-364947) long-term administration (e.g., 72 h).

Since WM266-4 cells have been subjected to a structural MT network re-modeling during the IMC process (Figure 5), vinblastine (*Vinca* alkaloid), a specific MT-destabilizing agent [317,318,319,320,321], was subsequently analyzed for its capacity to kill melanoma cells (Figure 9). Interestingly, WM266-4 presented a significant tolerance to vinblastine exposure for 24 h compared to WM115 at all administered doses, with the higher ones (50 and 100 μM) exhibiting the most important and clinically relevant differences (Figure 9B), which could be beneficially exploited for advanced disease therapeutic management. It may be that the re-organized, metastatic, MT cytoskeleton architecture renders WM266-4 cells (partly) resistant to vinblastine. Remarkably, *TUBB4A*/TBB4A (348.32), a brain-specific (tubulin) isotype [317], is exclusively detected in WM266-4 proteomic map (as its most abundantly expressed component) (Table S3 [35]), and thus it is herein suggested to essentially contribute to the acquisition of a metastatic MT network and a vinblastine (semi-)tolerance. Of note, downregulation of βIVa-tubulin (TBB4A) seems to be implicated in the increased sensitivity of lung cancer cells to the tubulin-binding agent vincristine (*Vinca* alkaloid) [322]. Hence, it could be the upregulation of TBB4A that endows BRAF^V600D^ metastatic melanoma cells with significant resistance to vinblastine via critical re-modeling of the MT cytoskeleton.

The capacity of cisplatin, a platinum-based drug that is widely used in cancer chemotherapy [260,261,323] to induce melanoma cell type-specific apoptotic responses (Figure 8) prompted us to next examine the survival profiling of WM115 and WM266-4 cells in response to 24 h cisplatin exposure (Figure 9). Both cell types were presented with almost identical viabilities for all administered drug doses. Of note, the higher cisplatin concentrations (50 and 100 μg/mL) proved highly efficient for either WM115 (primary) or WM266-4 (metastatic) melanoma cell elimination (Figure 9C), indicating their presumable utilization for clinical treatment of the disease. Intriguingly, despite its inability to trigger a strong (activated) caspase-3-mediated apoptotic program in WM266-4, but not WM115 cells (Figure 8), cisplatin at 50 and 100 μg/mL could markedly reduce the survival of both WM115 (primary) and WM266-4 (metastatic) melanoma cells in a rather identical manner (Figure 9C). This suggests the implication of non-apoptotic sub-routine(s) in cisplatin-induced death of BRAF^V600D^ primary and metastatic melanoma cells. Accordingly, cisplatin ototoxicity has been mechanistically associated, besides apoptosis, with activation of necroptosis, autophagy, and pyroptosis [324,325]. Thereby, agents that trigger autophagy (or necroptosis) could likely synergize with cisplatin to confer strong chemotherapeutic actions against LC3B-II^Low^ and BRAF^V600D^-positive metastatic melanomas.

Besides *Vinca* alkaloids (e.g., vinblastine or vincristine), paclitaxel (Taxol) is also categorized among the most successful MT-targeting chemotherapeutic drugs [318]. Paclitaxel is a specific MT-stabilizing agent that binds to the tubulin β-subunit with a nearly 1:1 stoichiometric ratio along the MT length [317,318,326,327,328]. Hence, paclitaxel was herein investigated for its cytotoxic activity against WM115 and WM266-4 cells (Figure 9). In contrast to vinblastine (Figure 9B), a 50 μM dose of paclitaxel, for 24 h exposure, was able to more efficiently kill the WM266-4 (metastatic) than the WM115 (primary) melanoma cells (Figure 9D), thus indicating its potential use in disease therapy. Although resistance to paclitaxel has been associated with (over-)expression of the *TUBB3*/TBB3 (βIII) (tubulin) isotype [317,329,330,331,332,333], TBB3 was herein detected to be rather equally, and strongly, expressed in WM115 (339.78) (Table S1) and WM266-4 (381.11) [35] (see also Table S10) melanoma cells, likely suggesting the engagement of TBB3-independent mechanism(s) of (primary) melanoma cell (partial) resistance to paclitaxel. However, *TUBA3C*/TBA3C (345.44) and *TUBA8*/TBA8 (239.26) (tubulin; α-subunit) isotypes are exclusively identified in the WM266-4 proteomic collection (Table S3, [35]), notably showing abundant expression patterns. Taken together, it may be that this TBA3C and/or TBA8 strong upregulation renders the β-subunits of WM266-4 MTs more receptive (e.g., accessible) to paclitaxel (high-affinity) binding compared to the WM115 respective ones. Systemic biomarkering for TBA3C and/or TBA8 isotypes and targeted drugging with paclitaxel may emerge as a novel and powerful strategy for BRAF^V600D^ metastatic melanoma therapy.

Since the tubulin family members *TUBB4A*/TBB4A (348.32), *TUBA3C*/TBA3C (345.44), and *TUBA8*/TBA8 (239.26) were abundantly expressed and exclusively identified in WM266-4 cells (Table S3, [35]), while the HIF1α protein could also be specifically detected in WM266-4, but not WM115 cells (Figure 7) (Table S3, [35]), we next in silico investigated the proximal and distal 5′-regulatory regions of *TUBB4A*, *TUBA3C*, and *TUBA8* genes for the “ACGTG” *cis*-element, which acts as a hypoxia-response element (HRE) that binds the HIF1(α) transcription factor [334]. Notably, putative HRE elements could be observed in all regulatory regions of the three genes, with *TUBB4A* and *TUBA8* carrying typical HRE sequences in their proximal promoter regions, respectively (data not shown), strongly suggesting the HIF1(α)-dependent transcriptional activation of *TUBB4A*, *TUBA3C*, and *TUBA8* genes, and thus their cognate protein product(s)-mediated re-modeling of the MT cytoskeleton network (e.g., for Invadopodia formation) during the IMC process of BRAF^V600D^ melanomagenesis.

**Figure 10:**
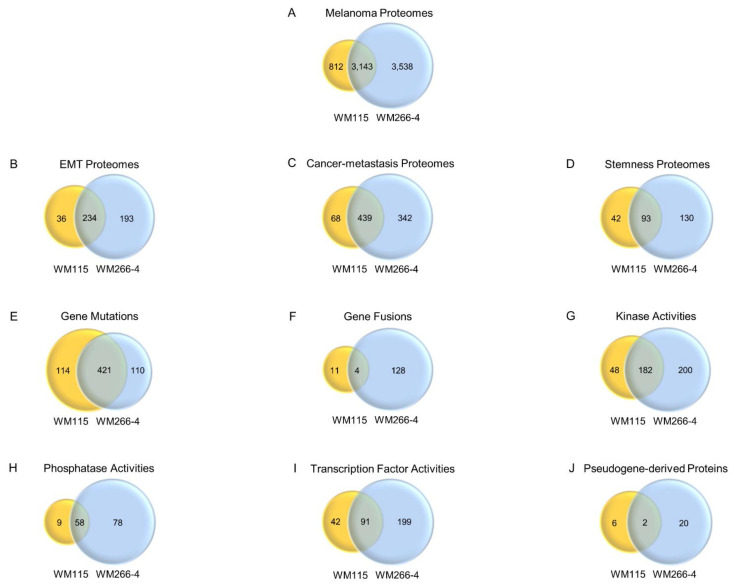
Proteomic numeration of human BRAF^V600D^-positive melanoma cells undergoing metastasis. (**A**–**J**) Venn diagrams describing the unique and common (overlapped circle areas) proteomic catalogues (protein collections) of WM115 (primary) (yellow-colored) and WM266-4 (metastatic) (blue-colored) BRAF^V600D^-dependent melanoma cells, according to their (**A**) total “Melanoma Proteomes” (nLC-MS/MS; UNIPROT [50]; this study and [35]), (**B**) “EMT Proteomes” (dbEMT; [58,59]), (**C**) “Cancer-metastasis Proteomes” (CMGene; [60]), (**D**) “Stemness Proteomes” (LifeMap Discovery; [61]), (**E**) “Gene Mutations” (DepMapPortal; [62,63,64,65]), (**F**) “Gene Fusions” (DepMapPortal; [62,63,64,65]), (**G**) “Kinase Activities” (GO_MF DAVID 6.8; [51,52,53]), (**H**) “Phosphatase Activities” (GO_MF DAVID 6.8; [51,52,53]), (**I**) “Transcription Factor Activities” (GO_MF DAVID 6.8; [51,52,53]), and (**J**) “Pseudogene-derived Proteins” (nLC-MS/MS; UNIPROT [50]; this study and [35]). (**D**) Venn diagram of shared genes expressed or selectively not expressed in human embryonic stem cells (hESCs) between WM115 (primary) and WM266-4 (metastatic) human melanoma cells. The genes involved in this analysis were obtained from LifeMap Discovery [61] and were manually curated from the literature and/or imported from high-throughput experiments. (**F**) Note the remarkably increased number (~11.5×) of unique “Gene Fusions” in WM266-4 (metastatic) (*n* = 128) (Table S19) compared to the respective WM115 (primary) (*n* = 11) (Table S18) melanoma cells ones.

Given the importance of mutant BRAF signaling in melanomagenesis [2,4,5,6,7,8,9,10,11,15,16,20,23], Sorafenib, a multi-kinase inhibitor [335,336,337,338,339], was tested next for BRAF^V600D^ melanoma-eradicating properties in WM115 and WM266-4 cells (Figure 9). The two cell types were presented with very similar survival patterns in response to the drug. Interestingly, 50 and 100 μM of sorafenib doses for 24 h exposure caused detrimental effects on WM115 (primary) and WM266-4 (metastatic) melanoma cell viabilities, with the highest concentration (100 μM) eliminating both melanoma cell cultures (Figure 9E). It seems that despite their different mutational signatures (Tables S4–S9) (see also Tables S18, S19, S28, and S29) and unique kinase family members (WM115: *n* = 48; WM266-4: *n* = 200) (Tables S2 and S3), (see also Tables S20 and S22, and Figure 10), WM115 and WM266-4 cells can be completely killed by clinically relevant doses of sorafenib (Figure 9E), thus unveiling the drug’s therapeutic value for successful management of BRAF^V600D^-dependent melanomagenesis.

The tolerance of WM266-4 cells to (activated) caspase-3-dependent apoptotic capacity of cisplatin (Figure 8) prompted us to further examine the presumable cytotoxic responses of WM115 and WM266-4 melanoma cells to epirubicin, an Anthracycline family member that blocks the catalytic activity of topoisomerase II and stabilizes DNA breaks, initiating cell death [340,341,342,343,344,345,346]. Surprisingly, WM266-4 melanoma cells presented a notable sensitivity to (24 h) treatment with epirubicin compared to the WM115 respective ones. Even at the lower drug doses of 1, 5, and 10 μM, WM266-4, but not WM115, cell viability was significantly reduced in a dose-dependent manner (Figure 9F). Altogether, it seems that epirubicin can specifically damage BRAF^V600D^-positive metastatic (e.g., WM266-4) melanoma cells at clinically low concentrations, thus opening a new therapeutic window for the advanced disease. Since autophagy inhibition augments the anti-cancer effect of epirubicin in breast cancer cells, while autophagy can protect them from epirubicin-induced apoptosis [340,347,348], it may be the compromised constitutive autophagy (LC3B-II^Low^) (Figure 8) that renders WM266-4 metastatic melanoma cells notably vulnerable to low doses of epirubicin (Figure 9F). Hence, combination of autophagy inhibitors (e.g., hydroxy-chloroquine) with epirubicin could prove therapeutically beneficial for (LC3B-II^High^) melanoma-suffering patients. Strikingly, besides elevated autophagy, increased levels of the SOX2 (NC) stem cell marker are also associated with anthracycline(s) resistance [340,349,350,351,352]. Given that WM115 cells carry elevated contents of nuclear SOX2 transcription factor (Figure 4), it may be the SOX2 upregulation that critically contributes to primary (e.g., WM115) melanoma cell resistance to low doses of epirubicin (Figure 9F). Altogether, systemic biomarkering for LC3B-II^Low^ (e.g., reduced constitutive autophagy), SOX2^Low^, and BRAF^V600D+^, and targeted drugging with low doses of epirubicin can likely emerge as a novel and promising approach for metastatic melanoma therapeutic management in the clinic.

## 4. Conclusions

The entire IMC process is exceptionally inefficient, with only a very small percentage of cells leaving primary tumor site(s) to form macroscopic metastasis(es) [37]. To better understand the IMC principles, it is imperative to promptly unveil the biological similarities and differences between primary tumors and their metastatic descendants.

Hence, by using the powerful pre-clinical platform of the primary melanoma cell line WM115 and the metastatic melanoma cell line WM266-4, both derived from the same patient, we have herein described the critical, proteomic-based, systemic biomarkering, and targeted drugging profiles to develop novel strategies for the successful management of advanced disease in the clinic. From the 812 and 3538 proteomic components uniquely identified in the WM115 and WM266-4 human melanoma cells, respectively (*n* = 3143 common) (Figure 10A and Tables S2, S3, and S10), 36 and 193 (*n* = 234 common) are recognized as “EMT program members” (Figure 10B and Tables S11–S13), 68 and 342 (*n* = 439 common) are categorized as “Cancer-metastasis proteins” (Figure 10C and Tables S13–S15), and 42 and 130 (*n* = 93 common) are classified as “Stemness-associated components” (Figure 10D and Tables S13, S16, and S17) (with the first and second, in a row, numerical value of each couple corresponding to WM115 and WM266-4 cells, respectively). Regarding their mutational loads, WM115 cells were specifically presented with 114 “Gene-mutation” and 11 “Gene-fusion” events, while the WM266-4 ones proved to exclusively carry 110 “Mutations” and 128 “Fusions” (*n* = 421 and *n* = 4 common, respectively) (Figure 10E,F and Tables S6, S7, S13, S18, and S19), strongly suggesting the major importance of “Gene-fusion” incidents to the IMC process in BRAF^V600D^ human melanoma(s). Similarly, WM266-4 cells were shown to contain 159 major chromosomal abnormalities including “Inversion-like”, “Deletion-like”, and “Duplication-like” incidents (Table S19) (“Cancer Dependency Map”—“Cancer Cell Line Encyclopedia”—Broad Institute; access day: 7 April 2021), thereby denoting the critical role(s) of chromosomal instability in IMC during BRAF^V600D^ human melanomagenesis.

Phosphorylation-dependent signaling homeostasis is reflected on the 48 “Kinase” and nine “Phosphatase” activity-bearing proteins in WM115, and on the 200 “Kinase” and 78 “Phosphatase” activity-carrying family members in the WM266-4 cells (*n* = 182 common kinases and *n* = 58 common phosphatases) (Figure 10G,H and Tables S13 and S20–S23), thus indicating the essential contribution of major signaling network(s) to melanoma cells undergoing IMC. Transcriptional activity is also upregulated in WM266-4 compared to WM115, since WM266-4 metastatic melanoma cells are shown to contain 199 “Transcription factor activity proteins”, while WM115 primary melanoma cells are presented with 42 respective proteomic components (*n* = 91 common) (Figure 10I, and Tables S13, S24, and S25). Remarkably, 6 and 20 “Pseudogene-derived (putative) proteins”, with presumably new functions, are uniquely recognized in the WM115 and WM266-4 cells, respectively (*n* = 2 common) (Figure 10J, and Tables S13, S26, and S27), revealing the pivotal roles of novel proteome members that are encoded by human pseudogenes in the IMC process during BRAF^V600D^-dependent melanomagenesis.

Most importantly, we herein demonstrate that IMC in mutant BRAF-positive melanoma environments seems to be typified by: (a) hybrid (intermediate) EMT/MET programs, (b) specific (NC-like) stemness sub-routines, (c) re-modeling of cytoskeleton architecture (to support migratory, invasion and colonization actions of tumor cells), (d) elevated constitutive activities of multiple signaling pathways, (e) HIF1α-driven gene expression, (f) attenuated (LC3B-II^Low^-driven) constitutive (basal) autophagy, (g) (partial) tolerance to genotoxicity, (h) (partial) resistance to (activated Caspase-3-directed) apoptosis, and (i) differential vulnerability to drug-induced cytotoxicity, with epirubicin exhibiting the strongest elimination of BRAF^V600D^ metastatic melanoma cells, dictating the necessity of its prompt utilization in advanced disease therapeutic strategies. Taken together, we herein propose an IMC-dependent protein immunophenotype signature (Table 1) that seems to hold strong biomarkering and drugging promise for mutant BRAF melanoma management in the clinic.

It seems that different combinations of IMC-associated programs can be activated in distinct melanoma cell sub-populations, productively collaborating to each other and mutually communicating with tumor micro-environment(s) to foster cancer evolution and engender sub-clonal heterogeneity toward strong chemoresistance and (macro-)metastasis. Because of the genetic and phenotypic heterogeneity, bulk tumors must contain diverse collections of cells that harbor different molecular signatures with variable levels of sensitivity to therapeutic treatments. However, mutational loads of BRAF^V600D^-dependent melanoma cells directly derived from cancer patients need to be carefully evaluated for disease-unrelated (d-un) SNPs (single nucleotide polymorphisms), whose exclusion from the oncogenic, clinically relevant, mutational signatures will numerically reduce the druggable tumorigenic targets of metastatic melanomas. Thereby, the gene-mutation unique profiles of WM115 (Table S6) and WM266-4 (Table S7) cells (Figure 10E) were herein processed to identify the respective d-un-SNP collections, and via their exclusion, to next provide novel and powerful biomarkering and drugging, combined, platforms for BRAF^V600D^ human malignant melanomas (Tables S28 and S29) (WM115: *n* = 77; WM266-4: *n* = 73).

## Figures and Tables

**Table 1:** IMC melanoma signaturing. Immunophenotyping of protein expression in WM115 (primary) and WM266-4 (metastatic) human melanoma cells.

		WM115			WM266-4		
	IF (phenotype)	Global Expression (100%)	Absence of Expression	Expression in Sub-Populations (%)	Global Expression (100%)	Absence of Expression	Expression in Sub-Populations (%)
Proteins							
Vimentin		+			+		
N-Cadherin				66		+	
E-Cadherin			+			+	
ZEB1				77			85
ZEB2				14			80
SLUG				63 (54: nuclear strong signal)			69 (3: nuclear strong signal)
p63α			+			+	
PRRX1				77			70
Keratin-5		+			+		
Keratin-8/18		+			+		
PDGFRβ				74			76
LOXL2				40			46
p-SMAD2		+			+		
SOX2		+					10
SOX9		+			+		
SOX10				2			11
p-STAT3				87			87
PCM1				83	+		
TRA1-60(S)		+		1.7 (strong signal)	+		
p-AKT		+			+		
p-GSK3β		+			+		
p-ERK1/2		+			+		
p-p38				90	+		
p-S6		+			+		
β-Catenin		+			+		
YAP				98			68
ATF4		+			+		
CHOP		+			+		
MCT1				97	+		
MCT4				95	+		
HIF1α			+				66
p-p53^Ser15^				7			7
p-p53^Ser37^				31			42
p-H2AX				48			44
a-Caspase-3			+			+	
DR5		+			+		
ATG12		+			+		

## Data Availability

Data are contained within the article or Supplementary Materials.

## Supplementary Materials

The following are available online at https://www.mdpi.com/article/10.3390/cancers13092024/s1; Figure S1. PDF file containing the uncropped Western blotting (ECL film) images, and the protein quantification values, in bar-chart format, analyzed in Figure 2C, Figure 7E and Figure 8G. Figure S2. Identification of the “E-x-x-C-V-x-L-x-x-x-D-x-x-x-[S/T]-x-x-[G/I]-[V/I]-x-[F/Y]-x-x-[S/T]” novel motif, exclusively recognized in KIAA0930 (K0930) and CEP44 proteins, via in silico engagement of the “MOTIF: Searching Protein Sequence Motifs – Genome Net” (MOTIF Search) bioinformatics tool. (A) “Motif Search” (against Sequence Database). (B) “Result of Pattern Search” (in Swiss-Prot). (C and D) “Motif in the Sequence” (red-colored fonts). (C) Human KIAA0930 (K0930). (D) Human CEP44. Table S1. Nano (n) LC-MS/MS-derived single protein library (*n* = 3955 proteome contents) of WM115 human primary melanoma cells (Microsoft Excel format file) (also, see Figure 10A), indicating, among others, the: (a) (UNIPROT) “Accession” (number), (b) “Description” (name), (c) (Mascot) “Score” (MS), (d) (Sequence) “Coverage”, (e) “Unique Peptides” (number) (*N* = 12,762 tryptic fragments), (f) “AAs” (amino acid number), (g) “MW” (molecular weight, in kDa) and (h) (calculated) “pI” (isoelectric point). Table S2. Proteomic catalogue (Microsoft Excel format file) of components specifically expressed in WM115 human primary melanoma cells (*n* = 812) (also, see Figure 10A), indicating, among others, the: (a) (UNIPROT) “Accession” (number), (b) “Description” (name), (c) (Mascot) “Score” (MS), (d) (Sequence) “Coverage”, (e) “Unique Peptides” (number), (f) “AAs” (amino acid number), (g) “MW” (molecular weight, in kDa) and (h) (calculated) “pI” (isoelectric point). Table S3. Collection of proteins (Microsoft Excel format file) exclusively identified in WM266-4 human metastatic melanoma cells (*n* = 3538) (also, see Figure 10A), indicating, among others, the: (a) (UNIPROT) “Accession” (number), (b) “Description” (name), (c) (Mascot) “Score” (MS), (d) (Sequence) “Coverage”, (e) “Unique Peptides” (number), (f) “AAs” (amino acid number), (g) “MW” (molecular weight, in kDa) and (h) (calculated) “pI” (isoelectric point). Table S4. Mutational signatures (single/double nucleotide polymorphisms, insertions and deletions) (Microsoft Excel format file) of WM115 human primary melanoma cells (*n* = 535 mutations) (also, see Figure 10E). “SNP”: Single Nucleotide Polymorphism. “DNP”: Double Nucleotide Polymorphism. “INS”: Insertion. “DEL”: Deletion. “Chr”: Chromosome. Table S5. Mutational signatures (single/double nucleotide polymorphisms, insertions and deletions) (Microsoft Excel format file) of WM266-4 human metastatic melanoma cells (*n* = 531 mutations) (also, see Figure 10E). “SNP”: Single Nucleotide Polymorphism. “DNP”: Double Nucleotide Polymorphism. “INS”: Insertion. “DEL”: Deletion. “Chr”: Chromosome. Table S6. Unique mutations (Microsoft Excel format file) of WM115 human primary melanoma cells (*n* = 114) (also, see Figure 10E). “SNP”: Single Nucleotide Polymorphism. “DNP”: Double Nucleotide Polymorphism. “INS”: Insertion. “DEL”: Deletion. “Chr”: Chromosome. Table S7. Unique mutations (Microsoft Excel format file) of WM266-4 human metastatic melanoma cells (*n* = 110) (also, see Figure 10E). Note the absence of Double Nucleotide Polymorphisms (DNPs) in the collection. “SNP”: Single Nucleotide Polymorphism. “INS”: Insertion. “DEL”: Deletion. “Chr”: Chromosome. Table S8. Fused-gene collection (Microsoft Excel format file) having been identified in WM115 human primary melanoma cells (*n* = 15) (also, see Figure 10F). Note the high frequency of “GT” and “AG” di-nucleotides at the “Left” and “Right” Breakpoints, respectively. Table S9. Fused-gene collection (Microsoft Excel format file) having been identified in WM266-4 human metastatic melanoma cells (*n* = 132) (also, see Figure 10F). Note the significantly higher number of gene-fusion incidents in metastatic (WM266-4) (*n* = 132), as compared to primary (WM115) (*n* = 15) (Table S8) melanoma cells. Table S10. Proteomic catalogue (Microsoft Excel format file) of common proteins being expressed both in WM115 (primary) and WM266-4 (metastatic) melanoma cells (*n* = 3143) (also, see Figure 10A). (UNIPROT) “Accession” (number), “Description” (name) and (Mascot) “Score” (MS) are indicated. Table S11. EMT program-specific collection (Microsoft Excel format file) of proteins exclusively expressed in WM115 human primary melanoma cells (*n* = 36) (also, see Figure 10B). Table S12. EMT program-specific collection (Microsoft Excel format file) of proteins exclusively expressed in WM266-4 human metastatic melanoma cells (*n* = 193) (also, see Figure 10B). Note the significantly higher number of EMT-related proteomic components in metastatic (WM266-4) (*n* = 193), as compared to primary (WM115) (*n* = 36) (Table S11) melanoma cells. Table S13. Proteomic catalogues (Microsoft Excel format file) of common proteins being expressed in both WM115 (primary) and WM266-4 (metastatic) human melanoma cells. 13B: “EMT Proteomes” (*n* = 234). 13C: “Cancer-metastasis Proteomes” (*n* = 439). 13D: “Stemness Proteomes” (*n* = 93). 13E: “Gene Mutations” (*n* = 421). 13F: “Gene Fusions” (*n* = 4). 13G: “Kinase Activities” (*n* = 182). 13H: “Phosphatase Activities” (*n* = 58). 13I: “Transcription Factor Activities” (*n* = 91). 13J: “Pseudogene-derived Proteins” (*n* = 2) (also, see Figure 10B–J). Note the notably high number of common proteomic contents classified in “EMT Proteomes” (*n* = 234), “Cancer-metastasis Proteomes” (*n* = 439) and “Gene Mutations” (*n* = 421) categories. Table S14. Collection (Microsoft Excel format file) of cancer-metastasis proteins uniquely identified in WM115 human primary melanoma cells (*n* = 68) (also, see Figure 10C). Table S15. Collection (Microsoft Excel format file) of cancer-metastasis proteins uniquely identified in WM266-4 human metastatic melanoma cells (*n* = 342) (also, see Figure 10C). Note the significantly higher number of cancer-metastasis catalogue contents in metastatic (WM266-4) (*n* = 342), as compared to primary (WM115) (*n* = 68) (Table S14) melanoma cells. Table S16. Catalogue (Microsoft Excel format file) of stemness-associated proteins that are exclusively expressed in WM115 human primary melanoma cells (*n* = 42) (also, see Figure 10D). Table S17. Catalogue (Microsoft Excel format file) of stemness-associated proteins that are exclusively expressed in WM266-4 human metastatic melanoma cells (*n* = 130) (also, see Figure 10D). Note the significantly higher number of stemness-associated components in metastatic (WM266-4) (*n* = 130), as compared to primary (WM115) (*n* = 42) (Table S16) melanoma cells. Table S18. Gene-fusion events (Microsoft Excel format file) specifically identified in WM115 human primary melanoma cells (*n* = 11) (also, see Figure 10F). Note the high frequency of “GT” and “AG” di-nucleotides at the “Left” and “Right” Breakpoints, respectively. Table S19. Gene-fusion events (Microsoft Excel format file) uniquely identified in WM266-4 human metastatic melanoma cells (*n* = 128) (also, see Figure 10F). Note the significantly higher number of gene-fusion incidents in metastatic (WM266-4) (*n* = 128), as compared to primary (WM115) (*n* = 11) (Table S18) melanoma cells (Sheet 1). Chromosomal abnormalities identified in WM266-4 human metastatic melanoma cells (*n* = 159) (Sheet 2). Note the remarkably high number of chromosomal aberrations in gene-intron sequence areas. “INV”: Inversion. “DEL”: Deletion. “DUP”: Duplication. “TRA”: Translocation. Table S20. Collection (Microsoft Excel format file) of kinases exclusively expressed in WM115 human primary melanoma cells (*n* = 48) (also, see Figure 10G). Table S21. Collection (Microsoft Excel format file) of phosphatases exclusively expressed in WM115 human primary melanoma cells (*n* = 9) (also, see Figure 10H). Table S22. Collection (Microsoft Excel format file) of kinases exclusively expressed in WM266-4 human metastatic melanoma cells (*n* = 200) (also, see Figure 10G). Note the significantly higher number of identified kinases in metastatic (WM266-4) (*n* = 200), as compared to primary (WM115) (*n* = 48) (Table S20) melanoma cells. Table S23. Collection (Microsoft Excel format file) of phosphatases exclusively expressed in WM266-4 human metastatic melanoma cells (*n* = 78) (also, see Figure 10H). Note the significantly higher number of identified phosphatases in metastatic (WM266-4) (*n* = 78), as compared to primary (WM115) (*n* = 9) (Table S21) melanoma cells. Table S24. Proteome catalogue (Microsoft Excel format file) of transcription factors uniquely identified in WM266-4 human metastatic melanoma cells (*n* = 199) (also, see Figure 10I). Note the high number of proteins carrying zinc-finger motifs. Table S25. Proteome catalogue (Microsoft Excel format file) of transcription factors uniquely identified in WM115 human primary melanoma cells (*n* = 42) (also, see Figure 10I). Note the significantly lower number of transcription factors in primary (WM115) (*n* = 42), as compared to metastatic (WM266-4) (*n* = 199) (Table S24) melanoma cells. Table S26. Collection (Microsoft Excel format file) of pseudogene-derived (putative) proteins specifically recognized in WM115 human primary melanoma cells (*n* = 6) (also, see Figure 10J). Note that the proteins with the highest Mascot Score (MS) (192.32 and 79.58) (also, see Table S2) belong to the HSP90 (molecular chaperon) protein family. Table S27. Collection (Microsoft Excel format file) of pseudogene-derived (putative) proteins exclusively recognized in WM266-4 human metastatic melanoma cells (*n* = 20) (also, see Figure 10J). Note the significantly higher number of identified pseudogene-derived (putative) proteins in metastatic (WM266-4) (*n* = 20), as compared to primary (WM115) (*n* = 6) (Table S26) melanoma cells. Table S28. Catalogue (Microsoft Excel format file) of mutations devoid of disease-unrelated (d-un) (or, hitherto characterized) SNPs specifically in WM115 human primary melanoma cells (*n* = 77). “SNP”: Single Nucleotide Polymorphism. “DNP”: Double Nucleotide Polymorphism. “INS”: Insertion. “DEL”: Deletion. “Chr”: Chromosome. Table S29. Catalogue (Microsoft Excel format file) of mutations devoid of disease-unrelated (d-un) (or, hitherto characterized) SNPs exclusively in WM266-4 human metastatic melanoma cells (*n* = 73). Note the absence of Double Nucleotide Polymorphisms (DNPs) in the collection. “SNP”: Single Nucleotide Polymorphism. “INS”: Insertion. “DEL”: Deletion. “Chr”: Chromosome.
