# Supplementary material for: From Proteomic Mapping to Invasion-Metastasis-Cascade Systemic Biomarkering and Targeted Drugging of Mutant BRAF-Dependent Human Cutaneous Melanomagenesis
Source: Cancers (Basel). 2021 Apr 22;13(9):2024. doi: 10.3390/cancers13092024 (PMC8122743; doi:10.3390/cancers13092024)
Supplement: Supplementary file 1 [file cancers-13-02024-s001.zip › Supplementary_Material_CANCERS_Revised_09_04_2021.pdf]

Research Article

From Proteomic Mapping to Invasion-Metastasis-Cascade Systemic Biomarkering and Targeted Drugging of mutant BRAF-dependent Human Cutaneous Melanomagenesis

Aikaterini F. Giannopoulou [1,¶], Athanassios D. Velentzas [1,¶], Athanasios K. Anagnostopoulos [2], Adamantia Agalou [3], Nikos C. Papandreou [1], Stamatia A. Katarachia [1], Dimitra G. Koumoundourou [1], Eumorphia G. Konstantakou [4], Vasiliki I. Pantazopoulou [5], Anastasios Delis [5], Maria T. Michailidi [1], Dimitrios Valakos [5], Dimitris Chatzopoulos [5], Popi Syntichaki [5], Vassiliki A. Iconomidou [1], Ourania E. Tsitsilonis [6], Issidora S. Papassideri [1], Gerassimos E. Voutsinas [7], Polydefkis Hatzopoulos [8], Dimitris Thanos [5], Dimitris Beis [3], Ema Anastasiadou [5], George Th. Tsangaris [2] and Dimitrios J. Stravopodis [1,*]

[1] Section of Cell Biology and Biophysics, Department of Biology, School of Science, National and Kapodistrian University of Athens (NKUA), 15701 Athens, Greece; aigiann@biol.uoa.gr (A.F.G.); tveletz@biol.uoa.gr (A.D.V.); npapand@biol.uoa.gr (N.C.P.); skatarachia@biol.uoa.gr (S.A.K.); dim.koum99@gmail.com (D.G.K.); marymichailidi@gmail.com (M.T.M.); veconom@biol.uoa.gr (V.A.I.); ipapasid@biol.uoa.gr (I.S.P.)
[2] Systems Biology Center, Biomedical Research Foundation of the Academy of Athens (BRFAA), 11527 Athens, Greece; atanagnost@bioacademy.gr (A.K.A.); gthtsangaris@bioacademy.gr (G.Th.T.)
[3] Center for Clinical, Experimental Surgery and Translational Research, Biomedical Research Foundation of the Academy of Athens (BRFAA), 11527 Athens, Greece; agalou@bioacademy.gr (A.A.); dbeis@bioacademy.gr (D.B.)
[4] Harvard Medical School, Massachusetts General Hospital Cancer Center (MGHCC), Charlestown, Boston, Massachusetts (MA) 02114, USA; ekonstantakou@mgh.harvard.edu
[5] Center of Basic Research, Biomedical Research Foundation of the Academy of Athens (BRFAA), 11527 Athens, Greece; vaspantazo@bioacademy.gr (V.I.P.); tdelis@bioacademy.gr (A.D.); dvalakos@bioacademy.gr (D.V.); dchatzop@bioacademy.gr (D.C.); synticha@bioacademy.gr (P.S.); thanos@bioacademy.gr (D.T.); anastasiadou@bioacademy.gr (E.A.)
[6] Section of Animal and Human Physiology, Department of Biology, School of Science, National and Kapodistrian University of Athens (NKUA), 15701 Athens, Greece; rtsitsil@biol.uoa.gr
[7] Laboratory of Molecular Carcinogenesis and Rare Disease Genetics, Institute of Biosciences and Applications (IBA), National Center for Scientific Research (NCSR) "Demokritos", 15310 Athens, Greece; mvoutsin@bio.demokritos.gr
[8] Department of Biotechnology, Agricultural University of Athens (AUA), 11855 Athens, Greece; phat@aua.gr
¶ These authors contributed equally to this work
* Correspondence: dstravop@biol.uoa.gr; Tel.: +30-210-727-4105; (D.J.S.)

Simple Summary

Despite the recent advances in human malignancy therapy, metastasis and chemoresistance remain the principal causes of cancer-derived deaths. Given the fatal forms of cutaneous metastatic melanoma, we herein employed primary (WM115) and metastatic (WM266-4) melanoma cells, both obtained from the same patient, to identify novel biomarkers and

therapeutic agents. Through state-of-the-art technologies, including deep proteome landscaping, immunofluorescence phenotyping and drug toxicity screening, we were able to describe new molecular programs, oncogenic drivers and drug regimens, controlling Invasion-Metastasis Cascade during $BRAF^{V600D}$-dependent melanomagenesis. It proved that proteomic navigation could foster the development of systemic biomarkering and targeted drugging for successful treatment of the advanced disease.

Abstract

Melanoma is classified among the most notoriously aggressive human cancers. Despite the recent progress, due to its propensity for metastasis and resistance to therapy, novel biomarkers and oncogenic molecular drivers are necessitated to be promptly identified for metastatic melanoma. Hence, by employing nano Liquid Chromatography – Tandem Mass Spectrometry deep proteomics technology, advanced Bioinformatics algorithms, Immunofluorescence, Western blotting, Wound healing protocols, Molecular modeling programs and MTT assays, we comparatively examined the respective proteomic contents of WM115 primary (n = 3,955 proteins) and WM266-4 metastatic (n = 6,681 proteins) melanoma cells. It proved that WM115 and WM266-4 cells have engaged hybrid Epithelial-to-Mesenchymal Transition/Mesenchymal-to-Epithelial Transition states, with TGF-β controlling their motility *in vitro*. They are characterized by different signatures of SOX-dependent neural crest-like stemness and distinct architectures of cytoskeleton network. Multiple signaling pathways have been already activated from the primary melanoma stage, whereas HIF1α, the major hypoxia-inducible factor, can be exclusively observed in metastatic melanoma cells. Invasion-Metastasis Cascade-specific sub-routines of activated Caspase-3-triggered apoptosis and LC3B-II-dependent constitutive autophagy were also unveiled. Importantly, WM115 and WM266-4 cells exhibited diverse drug response profiles, with Epirubicin holding considerable promise as beneficial drug for metastatic melanoma clinical management. It is the proteome navigation that enables systemic biomarkering and targeted drugging to open new therapeutic windows for the advanced disease.

Keywords: Biomarker; BRAF; Cancer; IMC; LC-MS/MS; Melanoma; Metastasis; Proteomics; WM115; WM266-4

Supplementary Materials

Tables

Table S1. Nano (n) LC-MS/MS-derived single protein library (n = 3,955 proteome contents) of WM115 human primary melanoma cells (Microsoft Excel format file) (also, see Figure 10A), indicating, among others, the: (a) (UNIPROT) "Accession" (number), (b) "Description" (name), (c) (Mascot) "Score" (MS), (d) (Sequence) "Coverage", (e) "Unique Peptides" (number) (N = 12,762 tryptic fragments), (f) "AAs" (amino acid number), (g) "MW" (molecular weight, in kDa) and (h) (calculated) "pI" (isoelectric point)

Table S2. Proteomic catalogue (Microsoft Excel format file) of components specifically expressed in WM115 human primary melanoma cells (n = 812) (also, see Figure 10A), indicating, among others, the: (a) (UNIPROT) "Accession" (number), (b) "Description" (name), (c) (Mascot) "Score" (MS), (d) (Sequence) "Coverage", (e) "Unique Peptides" (number), (f)

“AAs” (amino acid number), (g) “MW” (molecular weight, in kDa) and (h) (calculated) “pI” (isoelectric point)

Table S3. Collection of proteins (Microsoft Excel format file) exclusively identified in WM266-4 human metastatic melanoma cells (n = 3,538) (also, see Figure 10A), indicating, among others, the: (a) (UNIPROT) “Accession” (number), (b) “Description” (name), (c) (Mascot) “Score” (MS), (d) (Sequence) “Coverage”, (e) “Unique Peptides” (number), (f) “AAs” (amino acid number), (g) “MW” (molecular weight, in kDa) and (h) (calculated) “pI” (isoelectric point)

Table S4. Mutational signatures (single/double nucleotide polymorphisms, insertions and deletions) (Microsoft Excel format file) of WM115 human primary melanoma cells (n = 535 mutations) (also, see Figure 10E). “SNP”: Single Nucleotide Polymorphism. “DNP”: Double Nucleotide Polymorphism. “INS”: Insertion. “DEL”: Deletion. “Chr”: Chromosome

Table S5. Mutational signatures (single/double nucleotide polymorphisms, insertions and deletions) (Microsoft Excel format file) of WM266-4 human metastatic melanoma cells (n = 531 mutations) (also, see Figure 10E). “SNP”: Single Nucleotide Polymorphism. “DNP”: Double Nucleotide Polymorphism. “INS”: Insertion. “DEL”: Deletion. “Chr”: Chromosome

Table S6. Unique mutations (Microsoft Excel format file) of WM115 human primary melanoma cells (n = 114) (also, see Figure 10E). “SNP”: Single Nucleotide Polymorphism. “DNP”: Double Nucleotide Polymorphism. “INS”: Insertion. “DEL”: Deletion. “Chr”: Chromosome

Table S7. Unique mutations (Microsoft Excel format file) of WM266-4 human metastatic melanoma cells (n = 110) (also, see Figure 10E). Note the absence of Double Nucleotide Polymorphisms (DNPs) in the collection. “SNP”: Single Nucleotide Polymorphism. “INS”: Insertion. “DEL”: Deletion. “Chr”: Chromosome

Table S8. Fused-gene collection (Microsoft Excel format file) having been identified in WM115 human primary melanoma cells (n = 15) (also, see Figure 10F). Note the high frequency of “GT” and “AG” di-nucleotides at the “Left” and “Right” Breakpoints, respectively

Table S9. Fused-gene collection (Microsoft Excel format file) having been identified in WM266-4 human metastatic melanoma cells (n = 132) (also, see Figure 10F). Note the significantly higher number of gene-fusion incidents in metastatic (WM266-4) (n = 132), as compared to primary (WM115) (n = 15) (Table S8) melanoma cells

Table S10. Proteomic catalogue (Microsoft Excel format file) of common proteins being expressed both in WM115 (primary) and WM266-4 (metastatic) melanoma cells (n = 3,143) (also, see Figure 10A). (UNIPROT) “Accession” (number), “Description” (name) and (Mascot) “Score” (MS) are indicated

Table S11. EMT program-specific collection (Microsoft Excel format file) of proteins exclusively expressed in WM115 human primary melanoma cells (n = 36) (also, see Figure 10B)

Table S12. EMT program-specific collection (Microsoft Excel format file) of proteins exclusively expressed in WM266-4 human metastatic melanoma cells (n = 193) (also, see Figure 10B). Note the significantly higher number of EMT-related proteomic components in metastatic (WM266-4) (n = 193), as compared to primary (WM115) (n = 36) (Table S11) melanoma cells

Table S13. Proteomic catalogues (Microsoft Excel format file) of common proteins being expressed in both WM115 (primary) and WM266-4 (metastatic) human melanoma cells. 13B: "EMT Proteomes" (n = 234). 13C: "Cancer-metastasis Proteomes" (n = 439). 13D: "Stemness Proteomes" (n = 93). 13E: "Gene Mutations" (n = 421). 13F: "Gene Fusions" (n = 4). 13G: "Kinase Activities" (n = 182). 13H: "Phosphatase Activities" (n = 58). 13I: "Transcription Factor Activities" (n = 91). 13J: "Pseudogene-derived Proteins" (n = 2) (also, see Figures 10B-10J). Note the notably high number of common proteomic contents classified in "EMT Proteomes" (n = 234), "Cancer-metastasis Proteomes" (n = 439) and "Gene Mutations" (n = 421) categories

Table S14. Collection (Microsoft Excel format file) of cancer-metastasis proteins uniquely identified in WM115 human primary melanoma cells (n = 68) (also, see Figure 10C)

Table S15. Collection (Microsoft Excel format file) of cancer-metastasis proteins uniquely identified in WM266-4 human metastatic melanoma cells (n = 342) (also, see Figure 10C). Note the significantly higher number of cancer-metastasis catalogue contents in metastatic (WM266-4) (n = 342), as compared to primary (WM115) (n = 68) (Table S14) melanoma cells

Table S16. Catalogue (Microsoft Excel format file) of stemness-associated proteins that are exclusively expressed in WM115 human primary melanoma cells (n = 42) (also, see Figure 10D)

Table S17. Catalogue (Microsoft Excel format file) of stemness-associated proteins that are exclusively expressed in WM266-4 human metastatic melanoma cells (n = 130) (also, see Figure 10D). Note the significantly higher number of stemness-associated components in metastatic (WM266-4) (n = 130), as compared to primary (WM115) (n = 42) (Table S16) melanoma cells

Table S18. Gene-fusion events (Microsoft Excel format file) specifically identified in WM115 human primary melanoma cells (n = 11) (also, see Figure 10F). Note the high frequency of "GT" and "AG" di-nucleotides at the "Left" and "Right" Breakpoints, respectively

Table S19. Gene-fusion events (Microsoft Excel format file) uniquely identified in WM266-4 human metastatic melanoma cells (n = 128) (also, see Figure 10F). Note the significantly higher number of gene-fusion incidents in metastatic (WM266-4) (n = 128), as compared to primary (WM115) (n = 11) (Table S18) melanoma cells (Sheet 1). Chromosomal abnormalities identified in WM266-4 human metastatic melanoma cells (n = 159) (Sheet 2). Note the remarkably high number of chromosomal aberrations in gene-intron sequence areas. "INV": Inversion. "DEL": Deletion. "DUP": Duplication. "TRA": Translocation

Table S20. Collection (Microsoft Excel format file) of kinases exclusively expressed in WM115 human primary melanoma cells (n = 48) (also, see Figure 10G)

Table S21. Collection (Microsoft Excel format file) of phosphatases exclusively expressed in WM115 human primary melanoma cells (n = 9) (also, see Figure 10H)

Table S22. Collection (Microsoft Excel format file) of kinases exclusively expressed in WM266-4 human metastatic melanoma cells (n = 200) (also, see Figure 10G). Note the significantly higher number of identified kinases in metastatic (WM266-4) (n = 200), as compared to primary (WM115) (n = 48) (Table S20) melanoma cells

Table S23. Collection (Microsoft Excel format file) of phosphatases exclusively expressed in WM266-4 human metastatic melanoma cells (n = 78) (also, see Figure 10H). Note the

significantly higher number of identified phosphatases in metastatic (WM266-4) (n = 78), as compared to primary (WM115) (n = 9) (Table S21) melanoma cells

Table S24. Proteome catalogue (Microsoft Excel format file) of transcription factors uniquely identified in WM266-4 human metastatic melanoma cells (n = 199) (also, see Figure 10I). Note the high number of proteins carrying zinc-finger motifs

Table S25. Proteome catalogue (Microsoft Excel format file) of transcription factors uniquely identified in WM115 human primary melanoma cells (n = 42) (also, see Figure 10I). Note the significantly lower number of transcription factors in primary (WM115) (n = 42), as compared to metastatic (WM266-4) (n = 199) (Table S24) melanoma cells

Table S26. Collection (Microsoft Excel format file) of pseudogene-derived (putative) proteins specifically recognized in WM115 human primary melanoma cells (n = 6) (also, see Figure 10J). Note that the proteins with the highest Mascot Score (MS) (192.32 and 79.58) (also, see Table S2) belong to the HSP90 (molecular chaperon) protein family

Table S27. Collection (Microsoft Excel format file) of pseudogene-derived (putative) proteins exclusively recognized in WM266-4 human metastatic melanoma cells (n = 20) (also, see Figure 10J). Note the significantly higher number of identified pseudogene-derived (putative) proteins in metastatic (WM266-4) (n = 20), as compared to primary (WM115) (n = 6) (Table S26) melanoma cells

Table S28. Catalogue (Microsoft Excel format file) of mutations devoid of disease-unrelated (d-un) (or, hitherto characterized) SNPs specifically in WM115 human primary melanoma cells (n = 77). "SNP": Single Nucleotide Polymorphism. "DNP": Double Nucleotide Polymorphism. "INS": Insertion. "DEL": Deletion. "Chr": Chromosome

Table S29. Catalogue (Microsoft Excel format file) of mutations devoid of disease-unrelated (d-un) (or, hitherto characterized) SNPs exclusively in WM266-4 human metastatic melanoma cells (n = 73). Note the absence of Double Nucleotide Polymorphisms (DNPs) in the collection. "SNP": Single Nucleotide Polymorphism. "INS": Insertion. "DEL": Deletion. "Chr": Chromosome

Figures

Figure S1. PDF file containing the uncropped Western blotting (ECL film) images, and the protein quantification values, in bar-chart format, analyzed in Figures 2C, 7E and 8G.

Figure S2. Identification of the "E-x-x-C-V-x-L-x-x-x-D-x-x-x-[S/T]-x-x-[G/I]-[V/I]-x-[F/Y]-x-x-[S/T]" novel motif, exclusively recognized in KIAA0930 (K0930) and CEP44 proteins, via *in silico* engagement of the "MOTIF: Searching Protein Sequence Motifs – Genome Net" (MOTIF Search) bioinformatics tool. (**A**) "Motif Search" (against Sequence Database). (**B**) "Result of Pattern Search" (in Swiss-Prot). (**C** and **D**) "Motif in the Sequence" (red-colored fonts). (**C**) Human KIAA0930 (K0930). (**D**) Human CEP44.

Author Contributions: A.F.G., A.D.V., A.K.A., A.A. and D.B. performed the experiments. A.F.G., A.D.V., A.K.A., N.C.P., V.I.P., A.D., D.V., D.C., V.A.I., D.T., E.A., G.Th.T. and D.J.S. run the bioinformatics algorithms. A.F.G., A.D.V., N.C.P., E.G.K., V.I.P., A.D., D.V., D.C., V.A.I., D.T., D.B., E.A., G.Th.T. and D.J.S. contributed analysis tools. A.A., D.B. and D.J.S. provided biological material. S.A.K., D.G.K. and M.T.M. offered technical support. A.F.G., A.D.V.,

A.K.A., A.A., N.C.P., S.A.K., D.G.K., E.G.K., V.I.P., A.D., M.T.M., D.V., D.C., P.S., V.A.I., O.E.T., I.S.P., G.E.V., P.H., D.T., D.B., E.A., G.Th.T. and D.J.S. analyzed, and interpreted the data. D.J.S. obtained the funding, conceived the project, designed the experiments, coordinated, and supervised the study and wrote the manuscript. All authors read and approved the final manuscript.

Funding: Financial support was provided by: (a) the "European Social Fund UoA – MIS 375784"; European Union and Greek National Funds 2012-2015; "THALIS" Program and (b) the "Bodossaki Foundation" Donation Program 2013-2014, Athens, Greece, to D.J.S. Funders had no role, whatsoever, in study organization, experimental design, data collection, results interpretation, manuscript preparation and decision to publish.

Acknowledgements: D.J.S. would like to devote the present article to the memory of his beloved father, who suddenly died on January 3rd, 2021.

Conflicts of Interest: The authors declare no conflict of interest.
