# Supplementary figures and images for: From Proteomic Mapping to Invasion-Metastasis-Cascade Systemic Biomarkering and Targeted Drugging of Mutant BRAF-Dependent Human Cutaneous Melanomagenesis
Source: Cancers (Basel). 2021 Apr 22;13(9):2024. doi: 10.3390/cancers13092024 (PMC8122743; doi:10.3390/cancers13092024)

Figure 2C

Vimentin

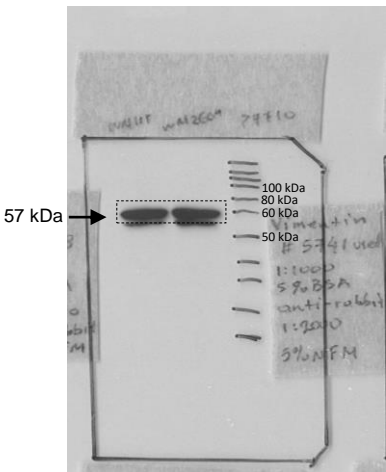

LOX

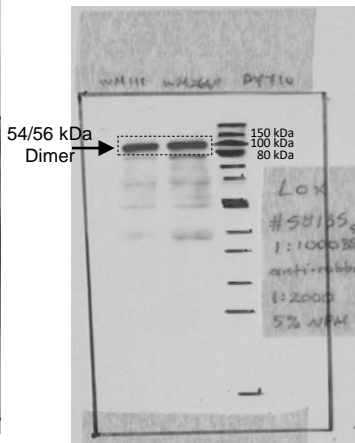

LOXL2

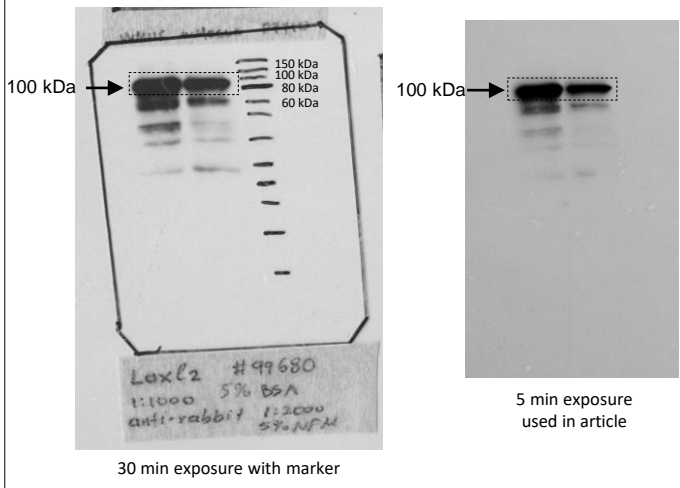

Pan-Actin

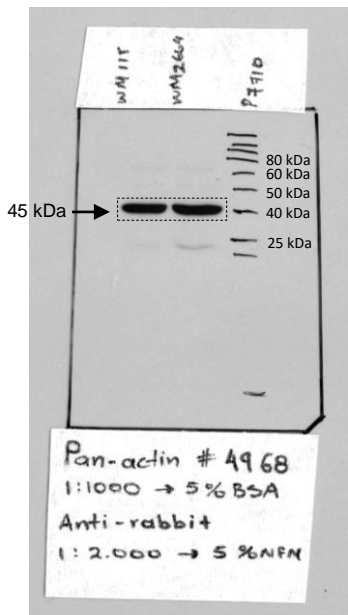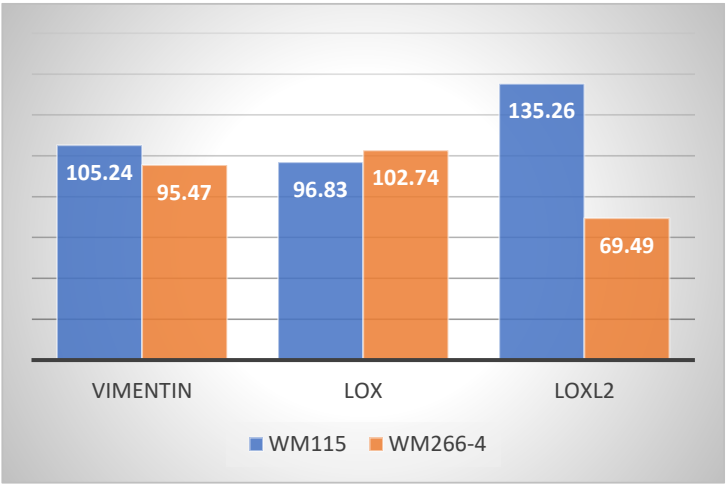

Figure 7E

HIF1α

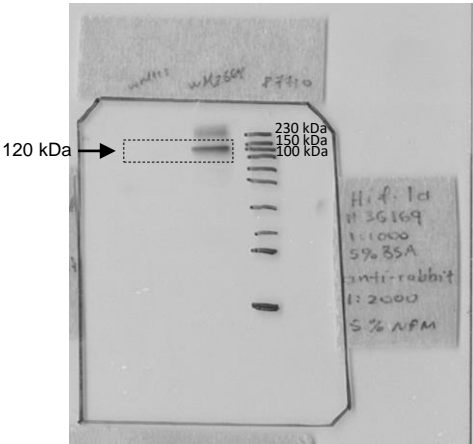

β-Tubulin

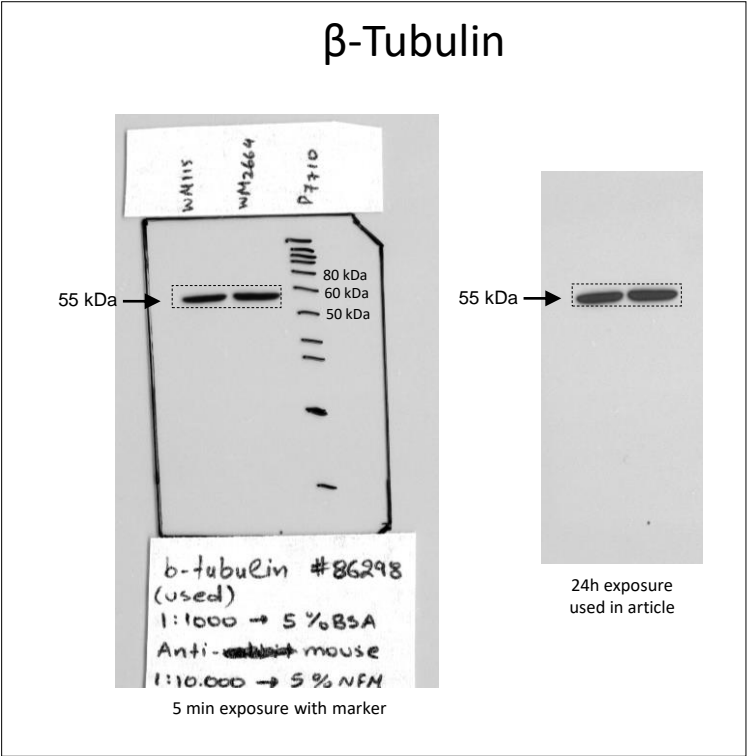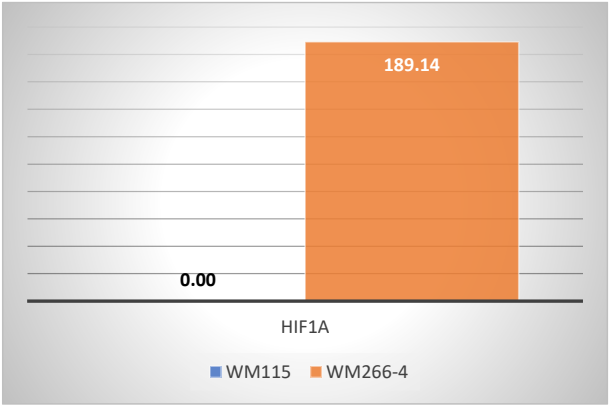

Figure 8G

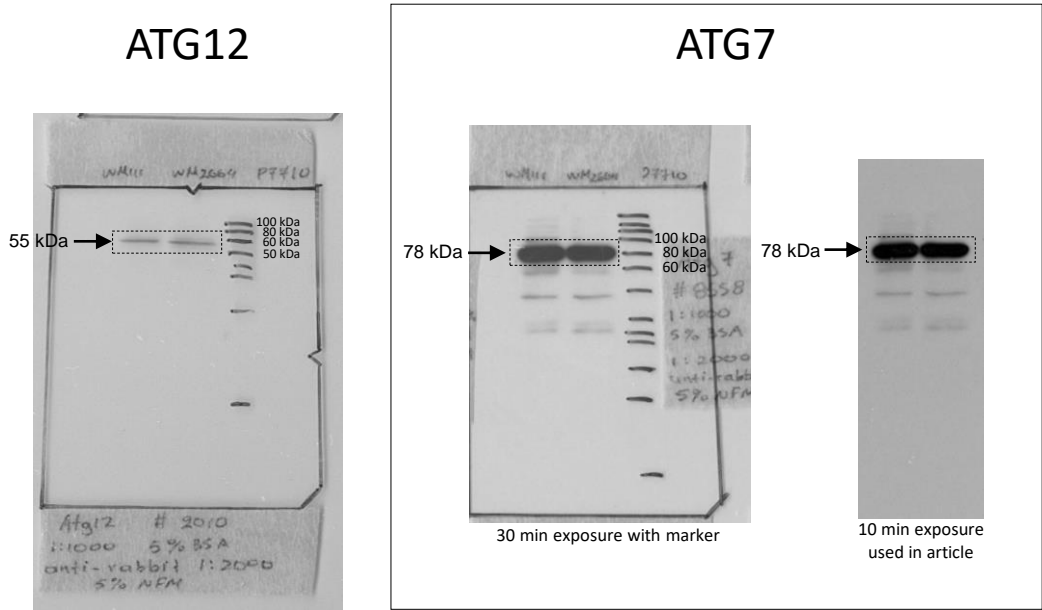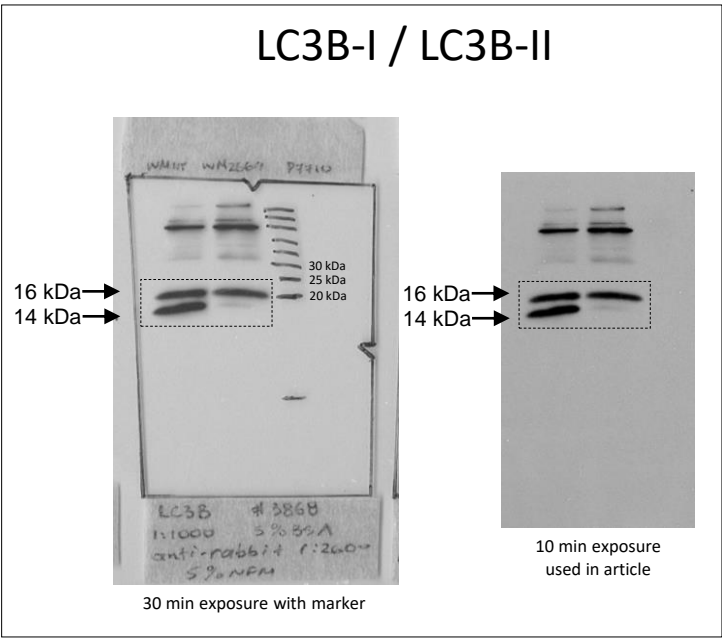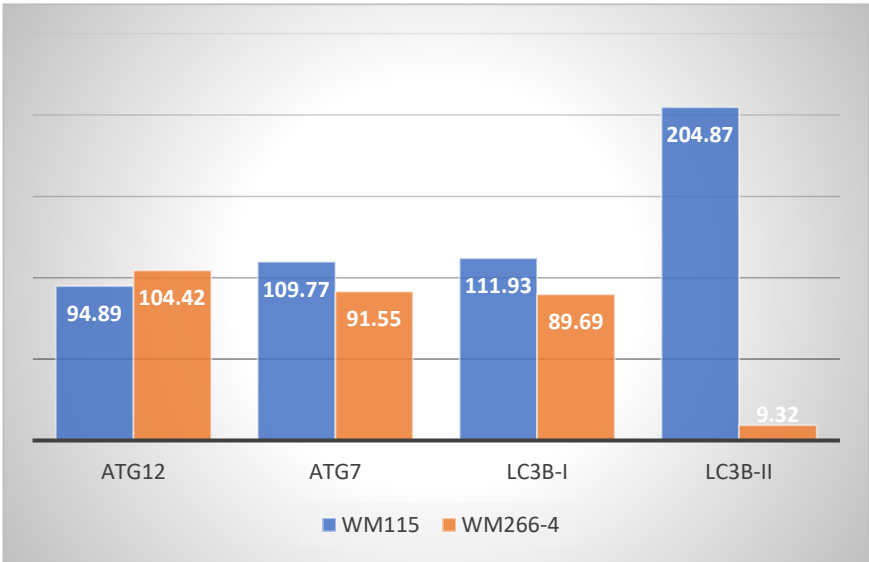

Supplement: Supplementary file 1 [file cancers-13-02024-s001.zip › Figure S1.pdf]

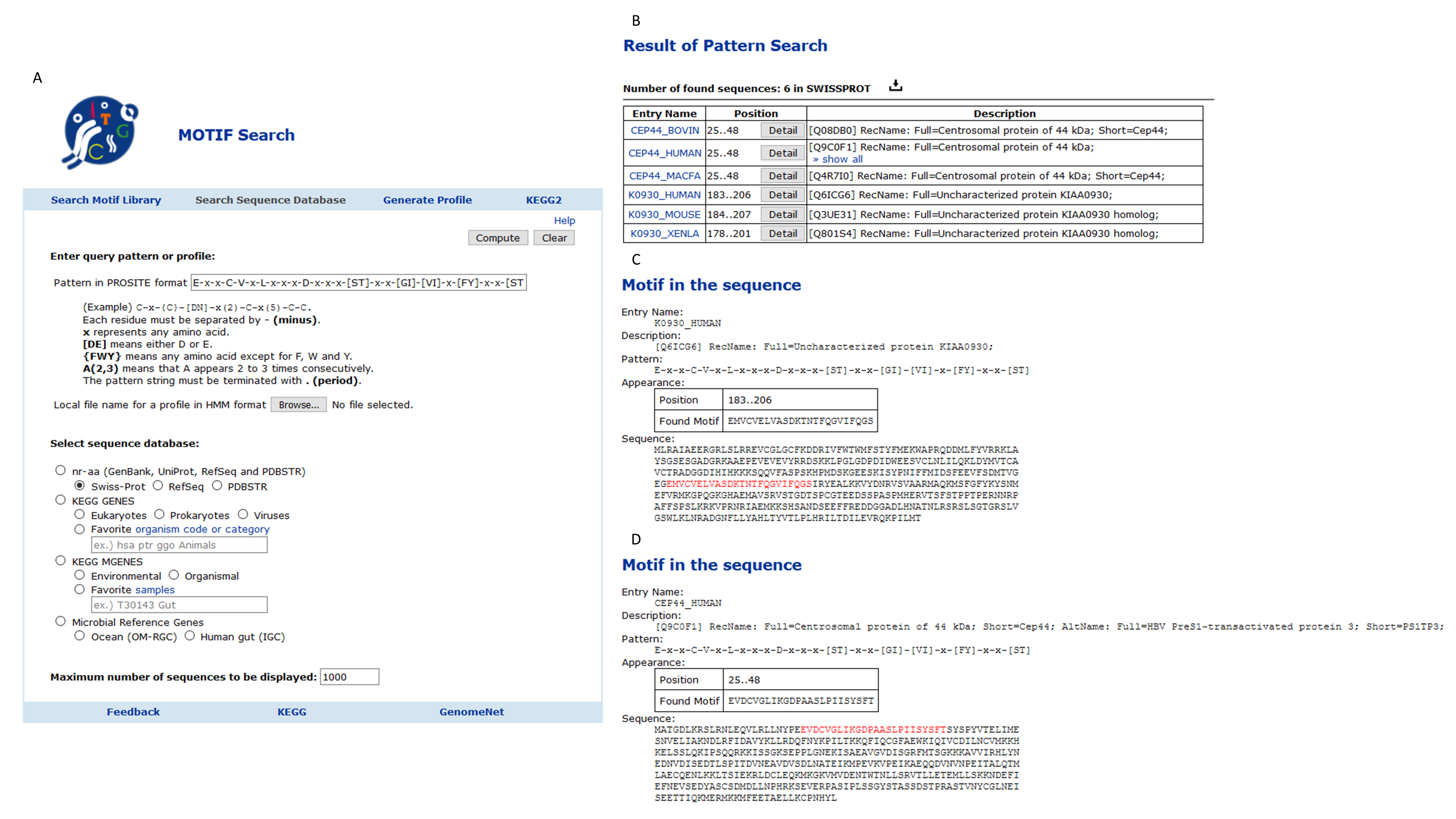

Supplement: Supplementary file 1 [file cancers-13-02024-s001.zip › Figure S2.tif]
